# Publisher Correction: Keratin-mediated hair growth and its underlying biological mechanism

**DOI:** 10.1038/s42003-022-04365-x

**Published:** 2022-12-22

**Authors:** Seong Yeong An, Hyo-Sung Kim, So Yeon Kim, Se Young Van, Han Jun Kim, Jae-Hyung Lee, Song Wook Han, Il Keun Kwon, Chul-Kyu Lee, Sun Hee Do, Yu-Shik Hwang

**Affiliations:** 1 Department of Maxillofacial Biomedical Engineering, College of Dentistry, Kyung Hee University, Seoul, 02447 Republic of Korea; 2 Department of Veterinary Clinical Pathology, College of Veterinary Medicine, Konkuk University, 120 Neungdong-ro, Gwangjin-gu, Seoul, 05029 Republic of Korea; 3 Department of Oral Microbiology, College of Dentistry, Kyung Hee University, Seoul, 02447 Republic of Korea; 4 KeraMedix Inc, # 204, Open Innovation Bld, Hongryeung Bio-Cluster, 117-3 Hoegi-ro, Dongdaemun-gu, Seoul, 02455 Republic of Korea; 5 Department of Dental Materials, College of Dentistry, Kyung Hee University, Seoul, 02447 Republic of Korea; 6 Headquarters of New Drug Development Support, Chemon Inc. 15F, Gyeonggi Bio Center, Cheongju, Gyeonggi-do 16229 Republic of Korea; 7 Present Address: Department of Dental Hygiene, College of Health Science, Cheongju University, Cheongju, 360-764 Republic of Korea; 8 Present Address: Terasaki Institute for Biomedical Innovation, Los Angeles, CA 90064 USA

**Keywords:** Biomaterials - cells, Stem-cell niche

Correction to: *Communications Biology* 10.1038/s42003-022-04232-9, published online 19 November 2022.

In this Article Sun Hee Do should have been denoted as a corresponding author.

This has now been corrected in the PDF and HTML versions of the Article.

