# Author Correction: Keratin-mediated hair growth and its underlying biological mechanism

**DOI:** 10.1038/s42003-022-04366-w

**Published:** 2022-12-22

**Authors:** Seong Yeong An, Hyo-Sung Kim, So Yeon Kim, Se Young Van, Han Jun Kim, Jae-Hyung Lee, Song Wook Han, Il Keun Kwon, Chul-Kyu Lee, Sun Hee Do, Yu-Shik Hwang

**Affiliations:** 1 Department of Maxillofacial Biomedical Engineering, College of Dentistry, Kyung Hee University, Seoul, 02447 Republic of Korea; 2 Department of Veterinary Clinical Pathology, College of Veterinary Medicine, Konkuk University, 120 Neungdong-ro, Gwangjin-gu, Seoul, 05029 Republic of Korea; 3 Department of Oral Microbiology, College of Dentistry, Kyung Hee University, Seoul, 02447 Republic of Korea; 4 KeraMedix Inc, # 204, Open Innovation Bld, Hongryeung Bio-Cluster, 117-3 Hoegi-ro, Dongdaemun-gu, Seoul, 02455 Republic of Korea; 5 Department of Dental Materials, College of Dentistry, Kyung Hee University, Seoul, 02447 Republic of Korea; 6 Headquarters of New Drug Development Support, Chemon Inc. 15F, Gyeonggi Bio Center, Cheongju, Gyeonggi-do 16229 Republic of Korea; 7 Present Address: Department of Dental Hygiene, College of Health Science, Cheongju University, Cheongju, 360-764 Republic of Korea; 8 Present Address: Terasaki Institute for Biomedical Innovation, Los Angeles, CA 90064 USA

**Keywords:** Biomaterials - cells, Stem-cell niche

Correction to: *Communications Biology* 10.1038/s42003-022-04232-9, published online 19 November 2022.

The Peer Review File that was originally published with the Article contained confidential data which the authors only intended to disclose to the peer reviewers. Figures containing the confidential data have now been redacted from pages 15, 22, 23, 38, and 45 of the Peer Review File.

