# Supplementary material for: Keratin-mediated hair growth and its underlying biological mechanism
Source: Commun Biol. 2022 Nov 19;5:1270. doi: 10.1038/s42003-022-04232-9 (PMC9675858; doi:10.1038/s42003-022-04232-9)
Supplement: Supplementary file 2 — Supplemental Information [file 42003_2022_4232_MOESM2_ESM.pdf]

SUPPLEMENTARY INFORMATION

Title. Keratin-mediated Hair Growth and its Underlying Biological Mechanism
Short title: Keratin-mediated Hair Growth

Authors

Seong Yeong An[1], Hyo-Sung Kim[2], So Yeon Kim[1ϕ], Se Young Van[1], Han Jun Kim[2#], Jae-Hyung Lee[1,3], Song Wook Han[4], Il Keun Kwon[5], Chul-Kyu Lee[6], Sun Hee Do[2]*, Yu-Shik Hwang[1]*

Affiliations

[1]Department of Maxillofacial Biomedical Engineering and Institute of Oral Biology, School of Dentistry, Kyung Hee University, Seoul 02447, Republic of Korea.
[2]Department of Veterinary Clinical Pathology, College of Veterinary Medicine, Konkuk University, 120 Neungdong-ro, Gwangjin-gu, Seoul 05029, Republic of Korea.
[3]Department of Life and Nanopharmaceutical Sciences, Kyung Hee University, Seoul 02447, Republic of Korea.
[4]KeraMedix Inc, # 204, Open Innovation Bld, Hongryeung Bio-Cluster, 117-3 Hoegi-ro, Dongdaemun-gu, Seoul 02455, Republic of Korea
[5]Department of Dental Materials and Institute of Oral Biology, School of Dentistry, Kyung Hee University, Seoul 02447, Republic of Korea.
[6]Headquarters of New Drug Development Support, Chemon Inc. 15F, Gyeonggi Bio Center, Gyeonggi-do, 16229, Republic of Korea,

[ϕ] Present Address: Department of Dental Hygiene, College of Health Science, Cheongju University, Cheongju 360-764, Republic of Korea
[#]Present Address: Terasaki Institute for Biomedical Innovation, Los Angeles, CA, 90064, United States

*To whom correspondence should be addressed at:

Sun Hee Do, PhD, * (**Co-corresponding author**)
Department of Veterinary Clinical Pathology, College of Veterinary Medicine, Konkuk University, 120 Neungdong-ro, Gwangjin-gu, Seoul 05029, Republic of Korea. Tel:+82-2-450-3706, Email: shdo@konkuk.ac.kr

Yu-Shik Hwang, PhD, * (**Co-corresponding author**)
Department of Maxillofacial Biomedical Engineering and Institute of Oral Biology, School of Dentistry, Kyung Hee University, 1 Hoegi-dong, Dongdaemun-gu, Seoul 02447, Republic of Korea. Tel: +82-2-961-2318, Email: yshwang@khu.ac.kr

SUPPLEMENTARY FIGURES

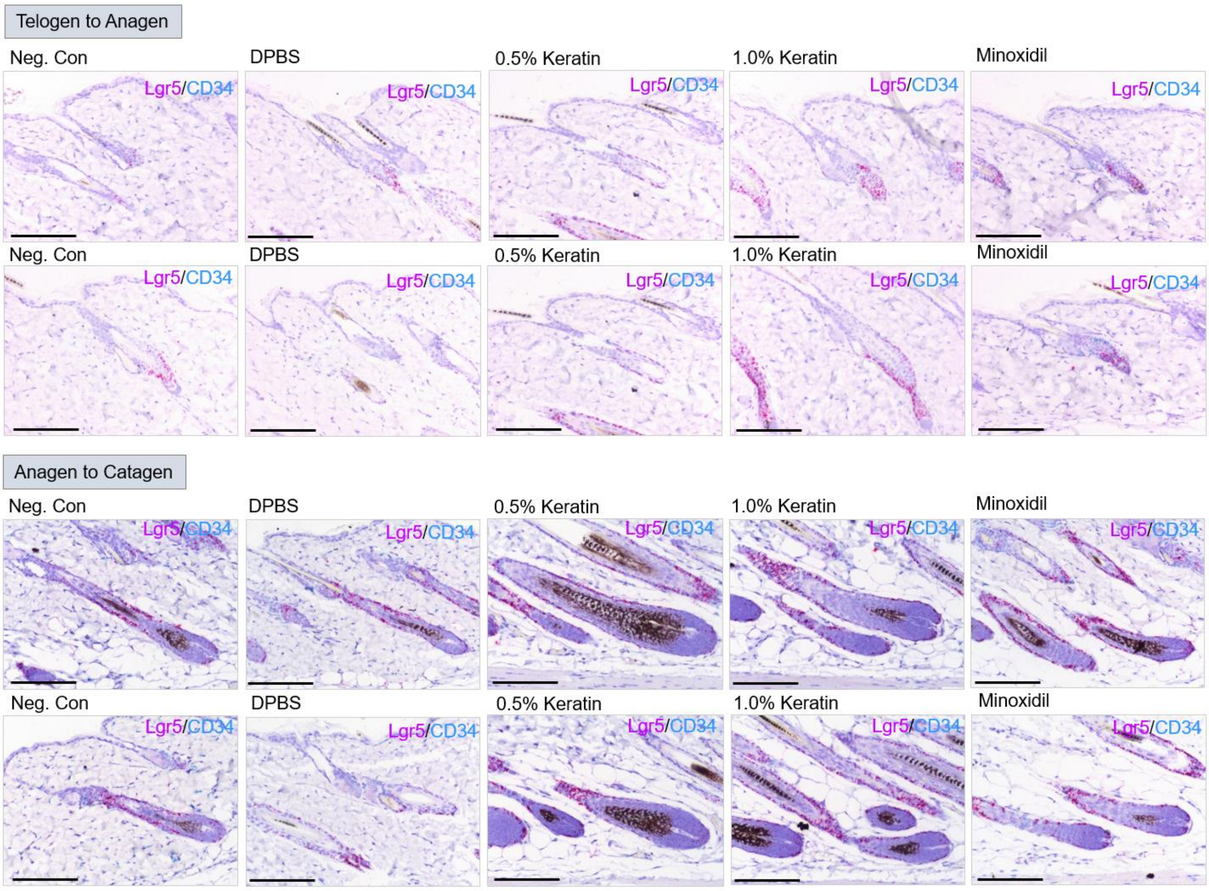

Supplementary Figure 1 | Intradermal injection of human hair-derived keratin induced the generation of Lgr5+ cell population. In situ hybridization analysis of the CD34 and Lgr5 mRNA of the back skin sections of C57BL/6 mice at 28 days after intradermal injection of keratin: Lgr 5, red; CD34, blue. Scale bars, 200μm.

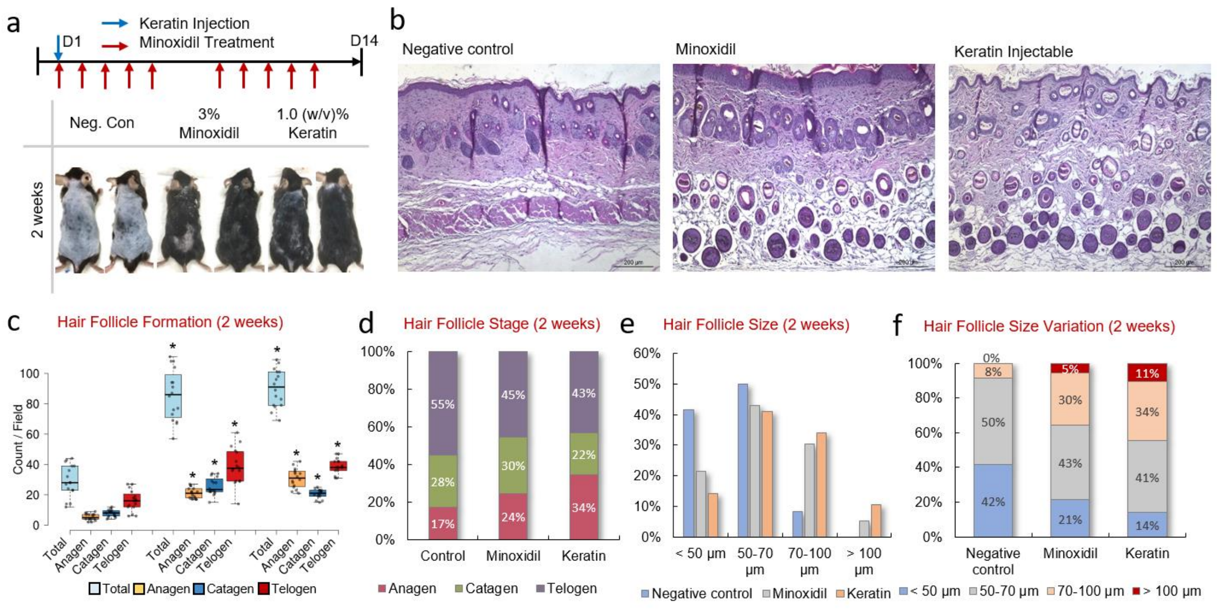

Supplementary Figure 2 | Intradermal injection of human hair-derived keratin induced hair follicle formation and improved hair growth, and *in vitro* keratin treatment induced DP condensation. a: Images of hair growth on the back skin of mice at 2 weeks after intradermal injection of keratin. b: Histological images of the back skin sections of mice at 2 weeks after intradermal injection of keratin. Scale bars, 200µm. c-f: Graphical representation and quantification of hair follicles; hair follicle formation (c), hair follicle stage (d), hair follicle size (e) and hair follicle size variations (f) in skin sections of mice (n = 16 sections, in 4 mice; mean ± standard deviation (s.d.)). *P,0.01, indicates a difference between control group and experimental groups.

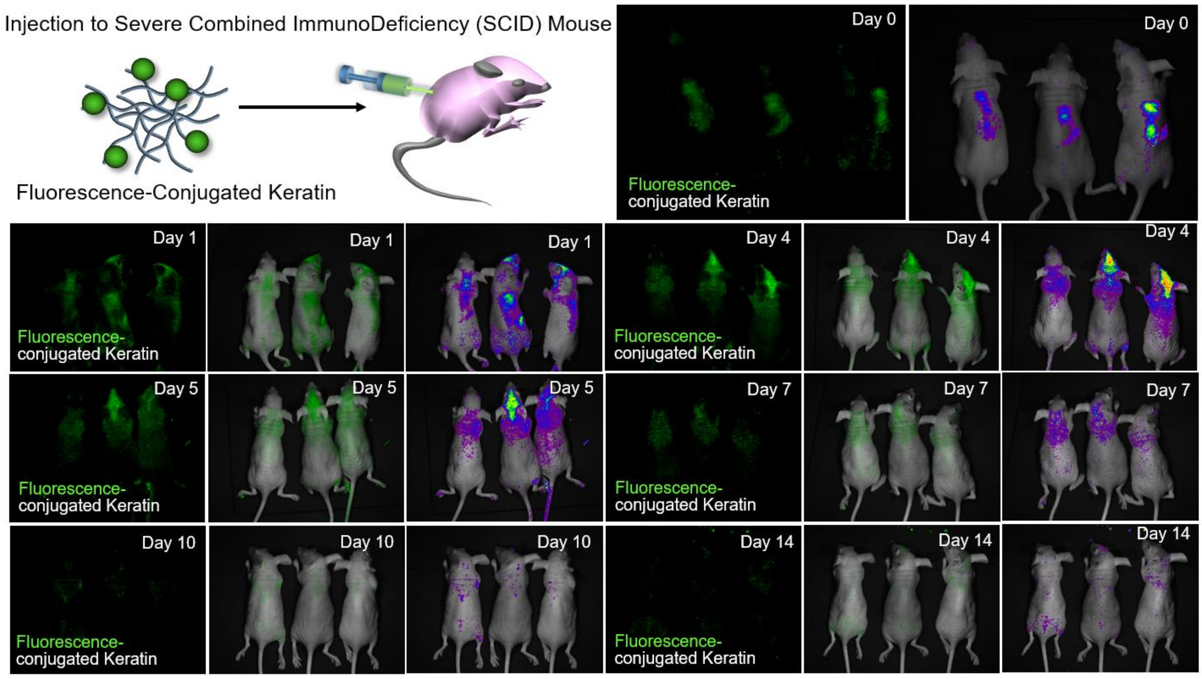

Supplementary Figure 3 | In vivo fluorescence imaging in SCID nude mice after intradermal injection of fluorescent dye-conjugated keratin at different time intervals.

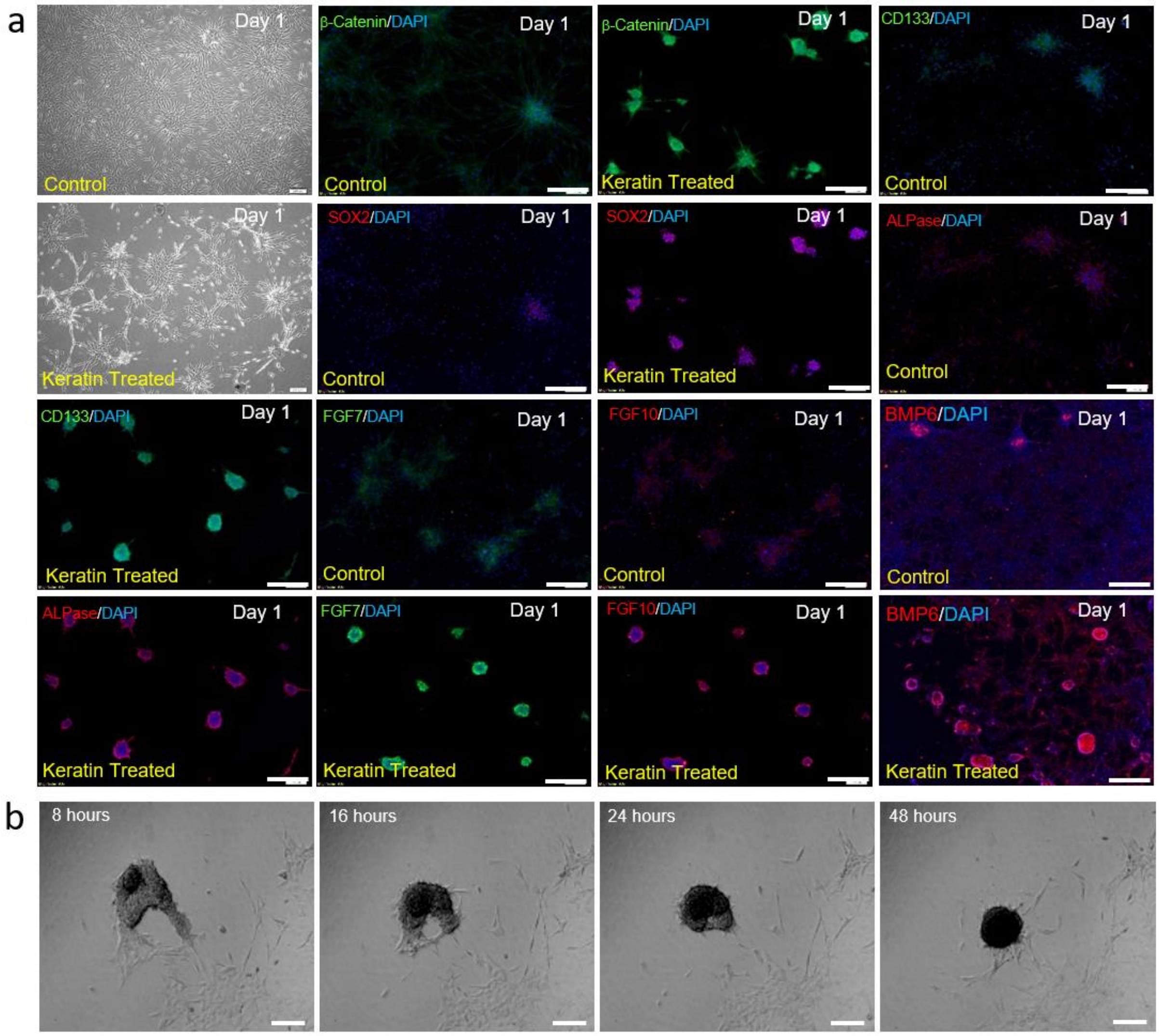

Supplementary Figure 4 | Hair keratin treatment upregulated DP cell property-related molecular expressions and induced DP cell condensation. a: Image of DP cell and DP cell condensation on day 1 after keratin treatment by immunofluorescent staining at low magnification; DAPI, blue; SOX2, ALPase, FGF10, BMP6, red; β-catenin, CD133, FGF7, green. Scale bars, 200μm. b: Time lapsed images of DP cell condensation in the presence of keratin. Scale bars, 100μm.

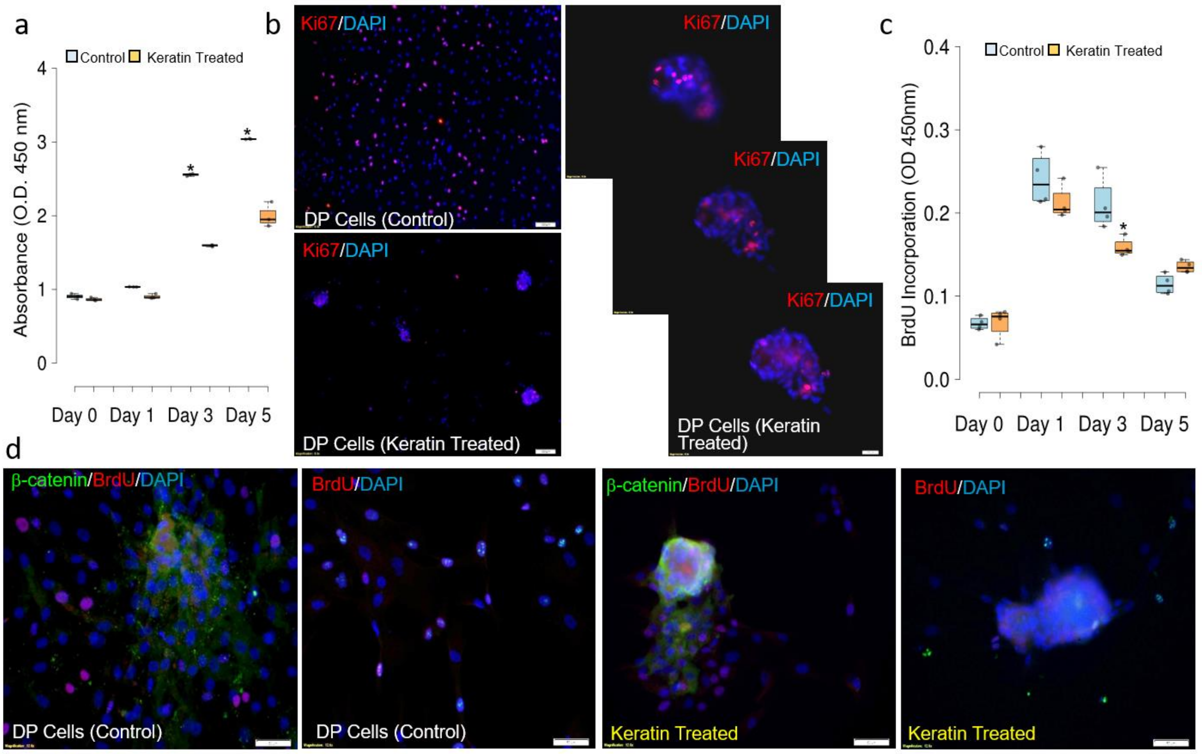

Supplementary Figure 5 | DP cell growth in the presence of hair keratin. a: Quantification of DP cell growth in the presence of keratin. *P,0.01, indicates a difference between control and keratin treated. (n=6; mean ± standard deviation (s.d.)). b: Image of DP cell and DP cell condensation on day 3 after keratin treatment by immunofluorescent staining; DAPI, blue; Ki67, red; Scale bars, 100μm. c: BrdU incorporation assay of DP cells in the presence of keratin. *P,0.01, indicates a difference between control and keratin treated. (n=4; mean ± standard deviation (s.d.)). s: Image of DP cell and DP cell condensation on day 3 after keratin treatment by immunofluorescent staining; DAPI, blue; BrdU, red; β-catenin, green; Scale bars, 50μm.

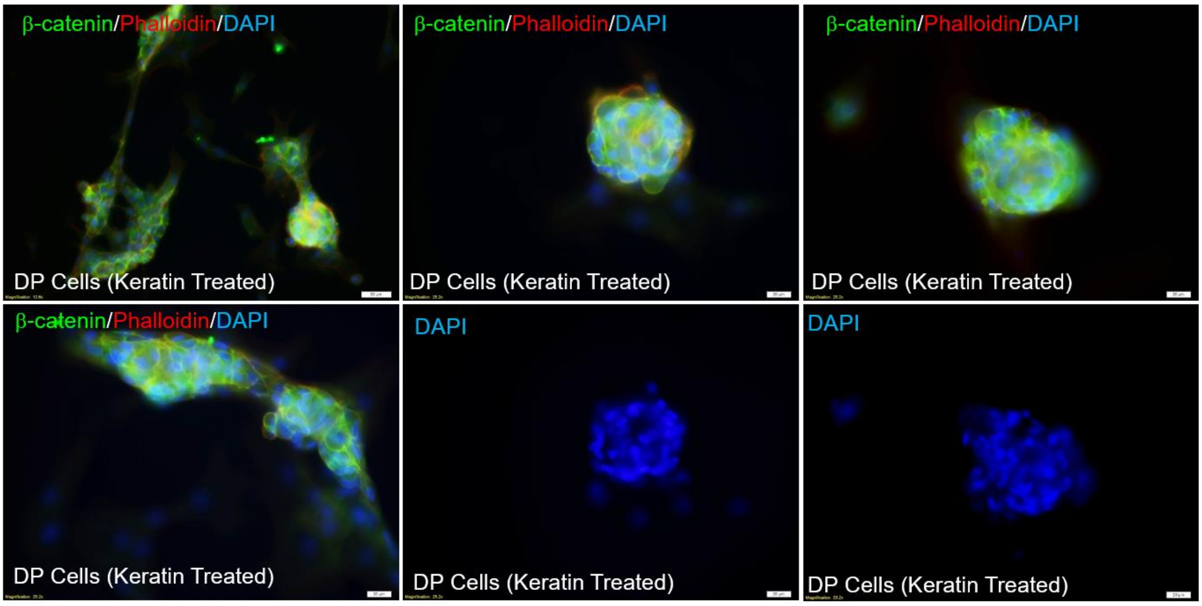

Supplementary Figure 6 | Magnified images of hair keratin-mediated DP cell condensation: Image of DP cell condensation on day 1 after keratin treatment by immunofluorescent staining; DAPI, blue; Phalloidin, red; β-catenin, green. Scale bars, 50μm and 20μm.

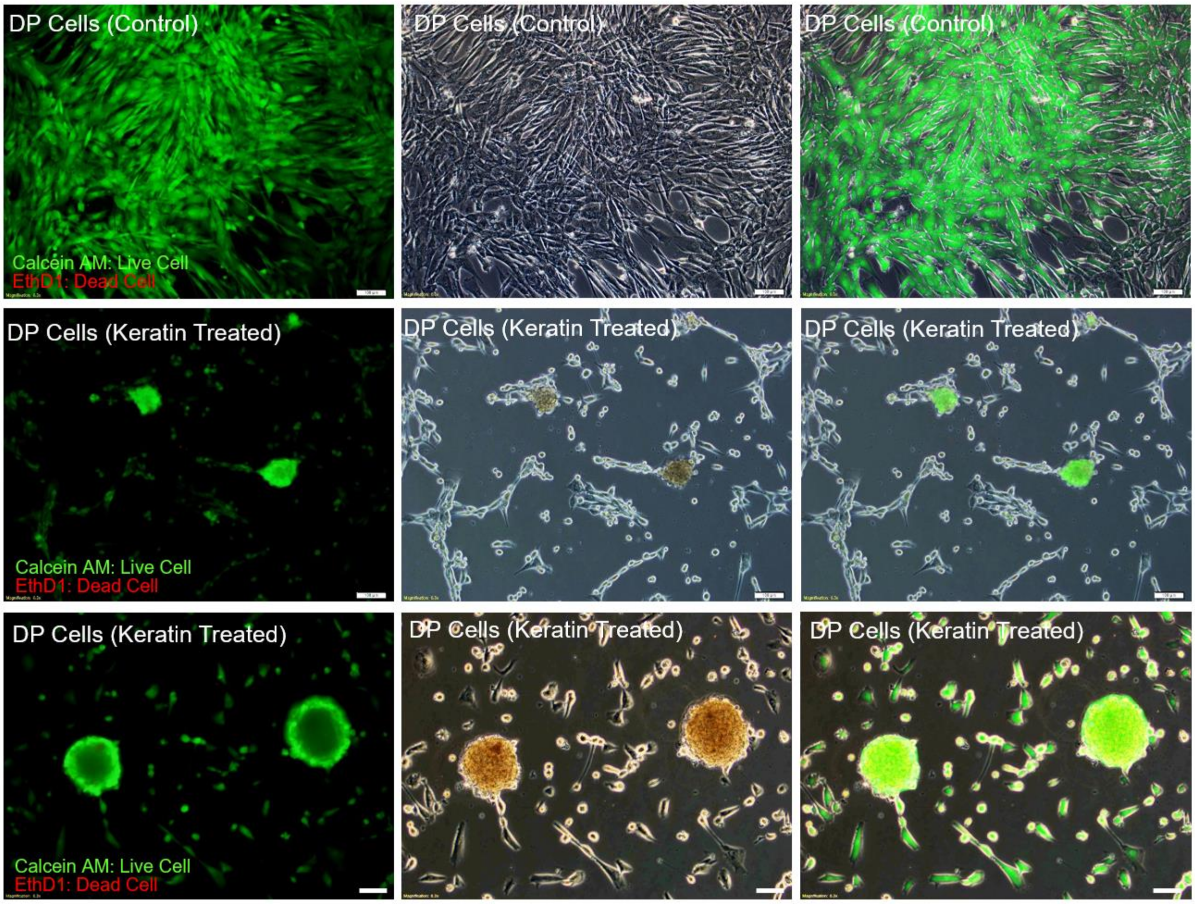

Supplementary Figure 7 | Hair keratin induces DP cell condensation and the condensed DP cell aggregates are highly viable: Images of DP cell and DP cell condensation on day 1 after keratin treatment by live/dead staining; Calcein AM, green; EthD1, red; Scale bars, 100μm.

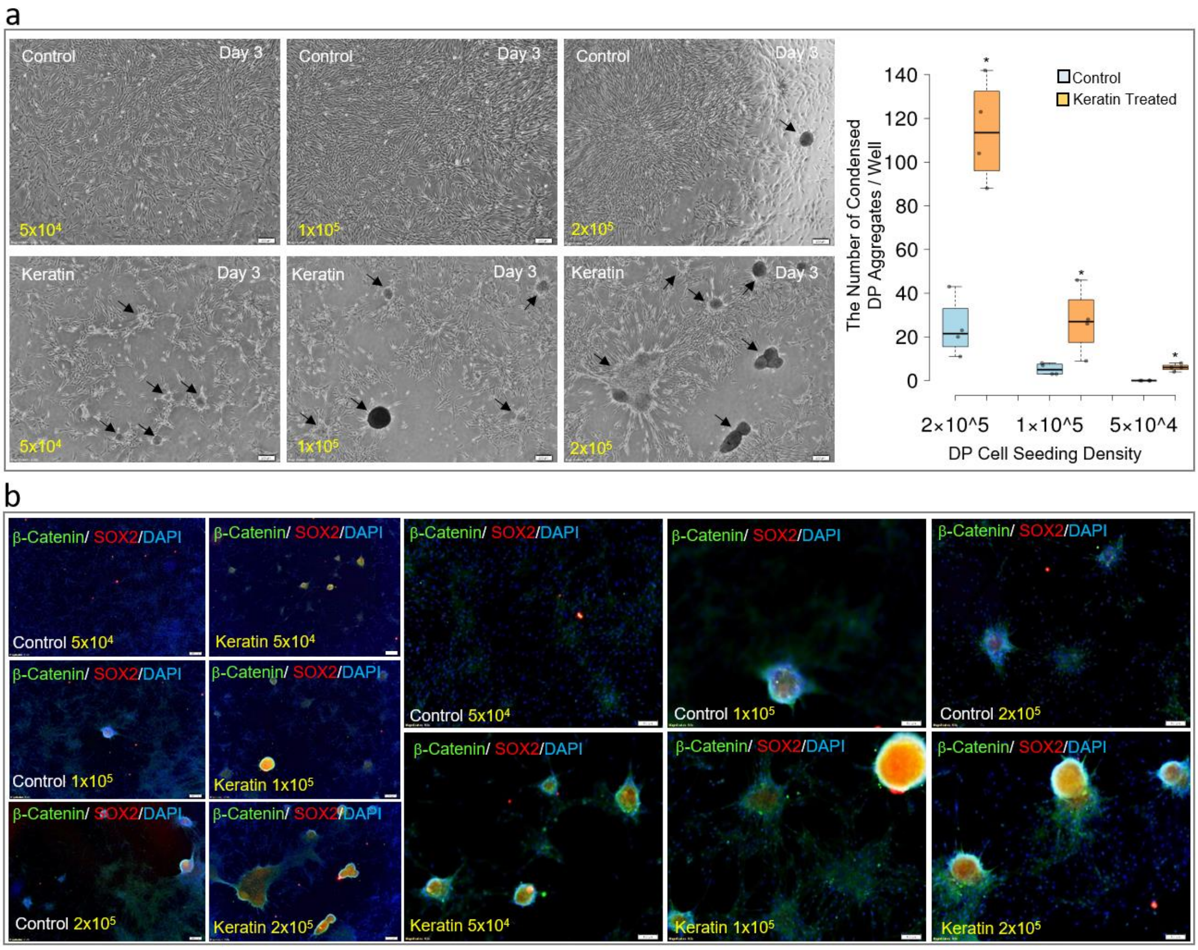

Supplementary Figure 8 | Hair keratin treatment induced DP cell condensation at different cell density. a: Images of DP cell condensation on day 3 after keratin treatment and graphical representation of DP cell condensation at different cell seeding densities. *P,0.01, indicates a difference between control and keratin treated. Scale bars, 200µm. (n=4; mean ± standard deviation (s.d.)). Arrows indicate the condensed DP cell aggregates. b: Images of DP cell condensation at different cell density in the presence of keratin by immunofluorescent staining; DAPI, blue; SOX2, red; β-catenin, green. Scale bars, 100µm.

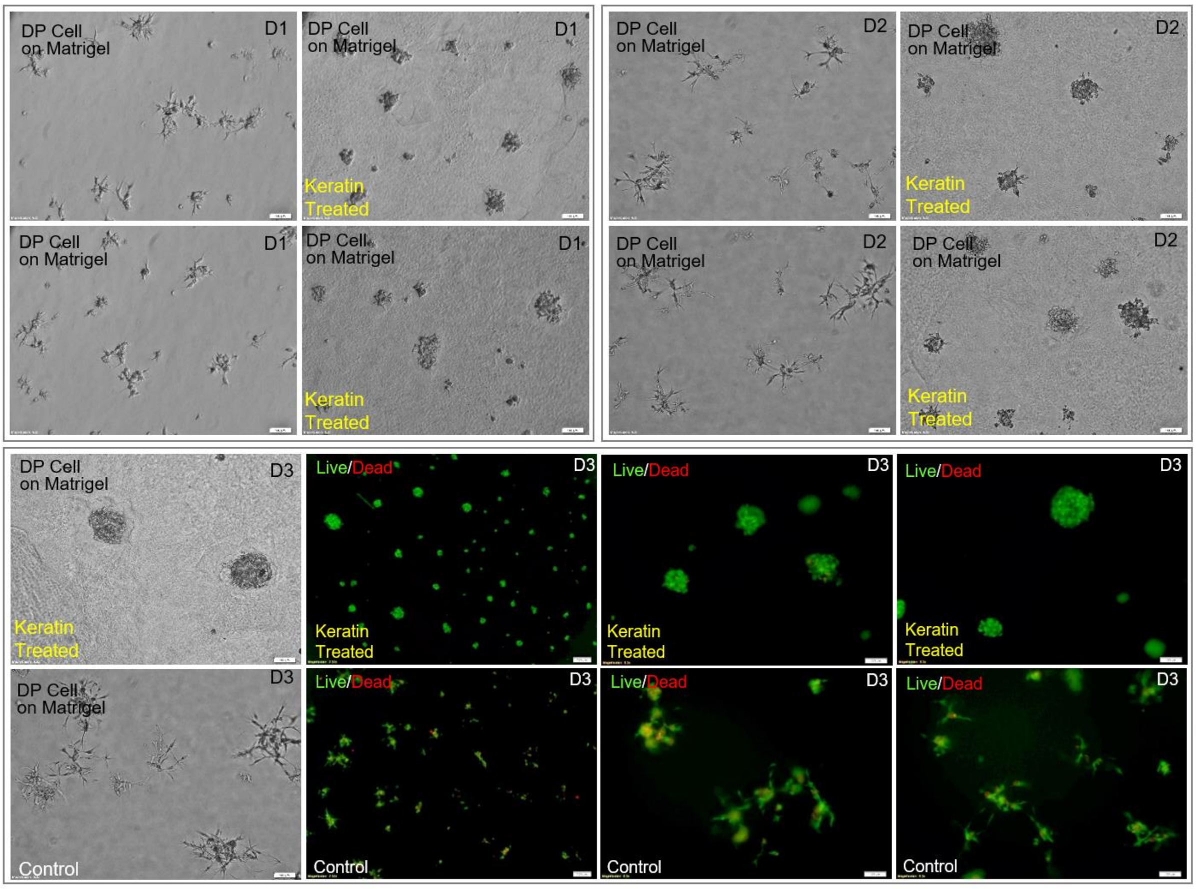

Supplementary Figure 9 | Hair keratin treatment induced DP cell condensation on matrigel. Images of DP cell condensation on matrigel in the presence of keratin and live/dead assay. Scale bars, 100μm.

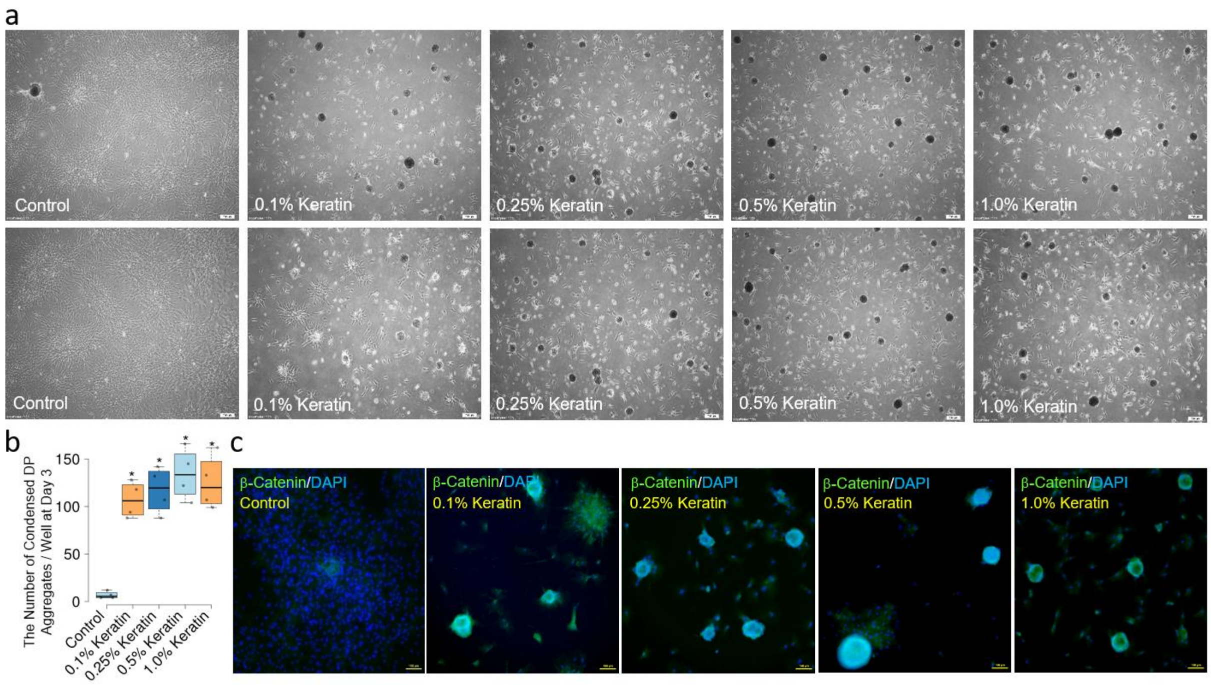

Supplementary Figure 10 | DP cell condensation at different keratin concentrations. a & b: Images of DP cell condensation on day 3 after treatment with different keratin concentrations, and graphical representation of DP cell condensation at different keratin concentrations. *P,0.01, indicates a difference between control and keratin treated. Scale bars, 200μm. (n=4; mean ± standard deviation (s.d.)). c: Images of DP cell condensation at different keratin concentrations by immunofluorescent staining; DAPI, blue; β-catenin, green. Scale bars, 100μm.

a

Upregulated Genes showing at least two fold Increase in Keratin Treated DP Cells

Gene name	term
C-C motif chemokine ligand 20(CCL20)	cell-cell signaling
hepatocyte growth factor(HGF)	positive regulation of cell migration
proprotein convertase subtilisin/kexin type 1(PCSK1)	cell-cell signaling
C-X-C motif chemokine ligand 5(CXCL5)	cell-cell signaling
TNF alpha induced protein 6(TNFAIP6)	cell-cell signaling
integrin subunit beta 8(ITGB8)	extracellular matrix organization
C-C motif chemokine ligand 7(CCL7)	cell-cell signaling
nicotinamide phosphoribosyl transferase(NAMPT)	cell-cell signaling
tachykinin precursor 1(TAC1)	cell-cell signaling
fibroblast growth factor 13(FGF13)	cell-cell signaling
C-C motif chemokine ligand 11(CCL11)	positive regulation of cell migration
interleukin 11(IL11)	cell-cell signaling
matrix metallopeptidase 9(MMP9)	Cell migration
C-X-C motif chemokine ligand 6(CXCL6)	cell-cell signaling
gap junction protein beta 2(GJB2)	cell-cell signaling
chondroitin sulfate N-acetylgalactosaminyltransferase 1(CSGALNACT1)	extracellular matrix organization
solute carrier family 7 member 11(SLC7A11)	Cell migration
angiopoietin 1(ANGPT1)	Cell migration
neuromedin B(NMB)	cell-cell signaling
laminin subunit gamma 3(LAMC3)	extracellular matrix organization
cell migration inducing hyaluronan binding protein(CEMIP)	positive regulation of cell migration
integrin subunit beta 3(ITGB3)	extracellular matrix organization
integrin subunit alpha V(ITGAV)	extracellular matrix organization
EGF like domain multiple 6(EGFL6)	extracellular matrix organization
Wnt family member 5A(WNT5A)	positive regulation of cell migration
neuropilin 2(NRP2)	positive regulation of cell migration
platelet derived growth factor D(PDGFD)	positive regulation of cell migration
integrin subunit alpha 2(ITGA2)	extracellular matrix organization
collagen type IV alpha 2 chain(COL4A2)	extracellular matrix organization
SAM and SH3 domain containing 1(SASH1)	positive regulation of cell migration
intercellular adhesion molecule 1(ICAM1)	extracellular matrix organization
copine 3(CPNE3)	positive regulation of cell migration
neuropilin 1(NRP1)	cell-cell signaling
endoplasmic reticulum oxidoreductase 1 beta(ERO1B)	extracellular matrix organization
integrin subunit alpha 8(ITGA8)	extracellular matrix organization
E74 like ETS transcription factor 3(ELF3)	extracellular matrix organization
matrix metallopeptidase 1(MMP1)	Cell migration
laeverin(LVRN)	cell-cell signaling
olfactomedin like 2A(OLFML2A)	extracellular matrix organization
integrin subunit alpha 4(ITGA4)	extracellular matrix organization
lumican(LUM)	extracellular matrix organization
collagen type IV alpha 1 chain(COL4A1)	extracellular matrix organization
C-C motif chemokine ligand 8(CCL8)	cell-cell signaling
WNT1 inducible signaling pathway protein 1(WISP1)	cell-cell signaling
semaphorin 6D(SEMA6D)	positive regulation of cell migration
collagen type IV alpha 4 chain(COL4A4)	extracellular matrix organization
ASH1 like histone lysine methyltransferase(ASH1L)	cell-cell signaling
semaphorin 5A(SEMA5A)	cell-cell signaling
sphingosine-1-phosphate receptor 1(S1PR1)	positive regulation of cell migration
fibroblast growth factor 2(FGF2)	extracellular matrix organization
vascular endothelial growth factor A(VEGFA)	positive regulation of cell migration
laminin subunit alpha 1(LAMA1)	extracellular matrix organization
ABI family member 3 binding protein(ABI3BP)	extracellular matrix organization
platelet derived growth factor receptor alpha(PDGFRA)	positive regulation of cell migration
nidogen 1(NID1)	extracellular matrix organization
ATPase copper transporting alpha(ATP7A)	extracellular matrix organization
nidogen 2(NID2)	extracellular matrix organization
insulin like growth factor 1(IGF1)	positive regulation of cell migration
neurofibromin 1(NF1)	extracellular matrix organization
transforming growth factor beta receptor 1(TGFBR1)	positive regulation of cell migration
TNF receptor superfamily member 11b(TNFRSF11B)	extracellular matrix organization
ADAM metallopeptidase domain 17(ADAM17)	positive regulation of cell migration
nephrocystin 3(NPHP3)	extracellular matrix organization
thrombospondin 1(THBS1)	extracellular matrix organization
integrin subunit beta 2(ITGB2)	extracellular matrix organization

b

Downregulated Genes showing at least two fold decrease in Keratin Treated DP Cells

Gene name	term
tubulin alpha 1b(TUBA1B)	cell division
cyclin B2(CCNB2)	regulation of cell cycle
tubulin alpha 1a(TUBA1A)	G1/M transition of mitotic cell cycle
TPX2, microtubule nucleation factor(TPX2)	mitotic nuclear division
inhibitor of DNA binding 3, HLH protein(ID3)	regulation of cell cycle
cyclin B1(CCNB1)	regulation of cell cycle
protein kinase, membrane associated tyrosine/threonine 1(PKMYT1)	regulation of cell cycle
forkhead box M1(FOXM1)	regulation of cell cycle
cell division cycle associated 3(CDCA3)	mitotic nuclear division
ubiquitin conjugating enzyme E2 C(UBE2C)	cell division
eukaryotic translation initiation factor 4E binding protein 1(EIF4EBP1)	positive regulation of mitotic cell cycle
aurora kinase B(AURKB)	mitotic nuclear division
SPC24, NDC80 kinetochore complex component(SPC24)	mitotic nuclear division
pituitary tumor-transforming 1(PTTG1)	mitotic nuclear division
asparagine synthetase (glutamine-hydrolyzing)(ASNS)	positive regulation of mitotic cell cycle
baculoviral IAP repeat containing 5(BIRC5)	mitotic nuclear division
polo like kinase 1(PLK1)	regulation of cell cycle
cell division cycle 20(CDC20)	mitotic nuclear division
growth arrest and DNA damage inducible beta(GADD45B)	regulation of cell cycle
TMF1-regulated nuclear protein 1(TRNP1)	regulation of cell cycle
tubulin beta 3 class III(TUBB3)	mitotic nuclear division
MX dynamin like GTPase 2(MX2)	regulation of cell cycle

Supplementary Figure 11 | Differentially expressed genes in DP cells upon on the keratin treatment. RNA-Seq data were acquired from the DP cells in the absences and cell in the presence of keratin. Differential expression test has been performed using DESeq2 method and the differentially expressed genes were defined as those with changes of at least 2-fold between samples at a false discovery rate (FDR) of 5%. a: Upregulated genes in the DP cells treated with keratin. b: Downregulated genes in the DP cells treated with keratin.

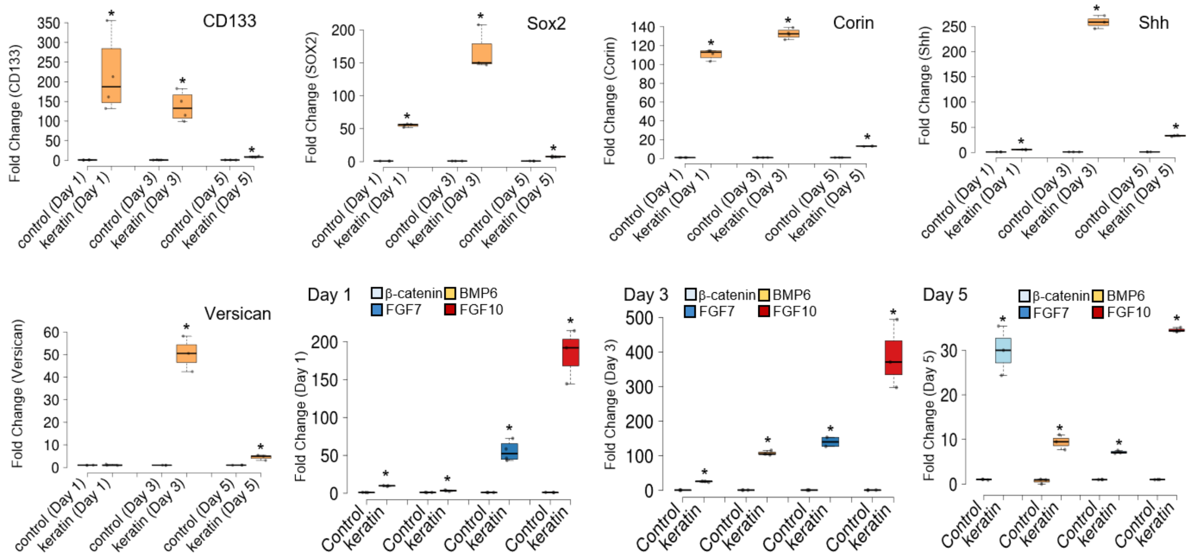

Supplementary Figure 12 | Hair keratin treatment upregulated DP cell property-related gene expressions and induced DP cell condensation. Graphical representation of realtime-qPCR analysis for *CD133*, *SOX2*, *Corin*, *SHH*, *Versican*, *β-catenin*, *BMP6*, *FGF7* and *FGF10* mRNA expressions in DP cell culture on day 1, 3 and 5 after keratin treatment. *P,0.01, indicates a difference between control and keratin treated. (n=4; mean ± standard deviation (s.d.)).

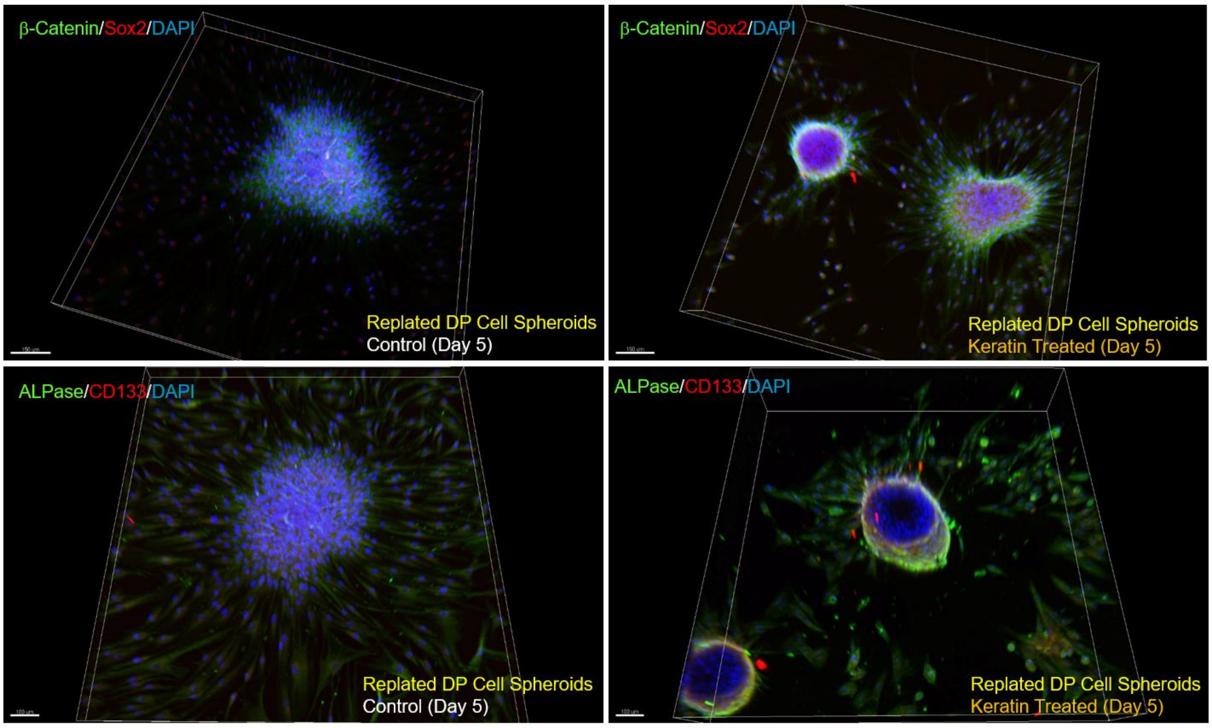

Supplementary Figure 13 | Confocal microscopic images of the replated DP cell spheroids in the presence of keratin or not: Image of the replated DP cell spheroids on day 5 after keratin treatment by immunofluorescent staining; DAPI, blue; SOX2, CD133, red; β-catenin, ALPase green. Scale bars, 150μm.

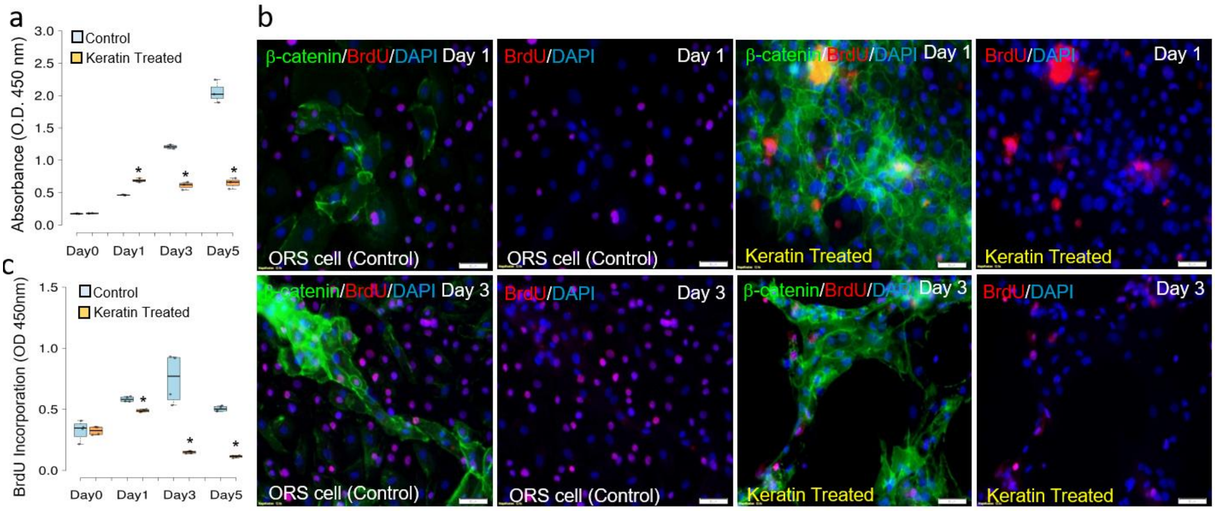

Supplementary Figure 14 | ORS cell growth in the presence of hair keratin. a: Quantification of ORS cell growth in the presence of keratin. *P,0.01, indicates a difference between control and keratin treated. (n=6; mean ± standard deviation (s.d.)). b: Image of ORS cells on day 1 and 3 after keratin treatment by immunofluorescent staining; DAPI, blue; BrdU, red; β-catenin, green. Scale bars, 50μm. c: BrdU incorporation assay of ORS cells in the presence of keratin. *P,0.01, indicates a difference between control and keratin treated. (n=4; mean ± standard deviation (s.d.)).

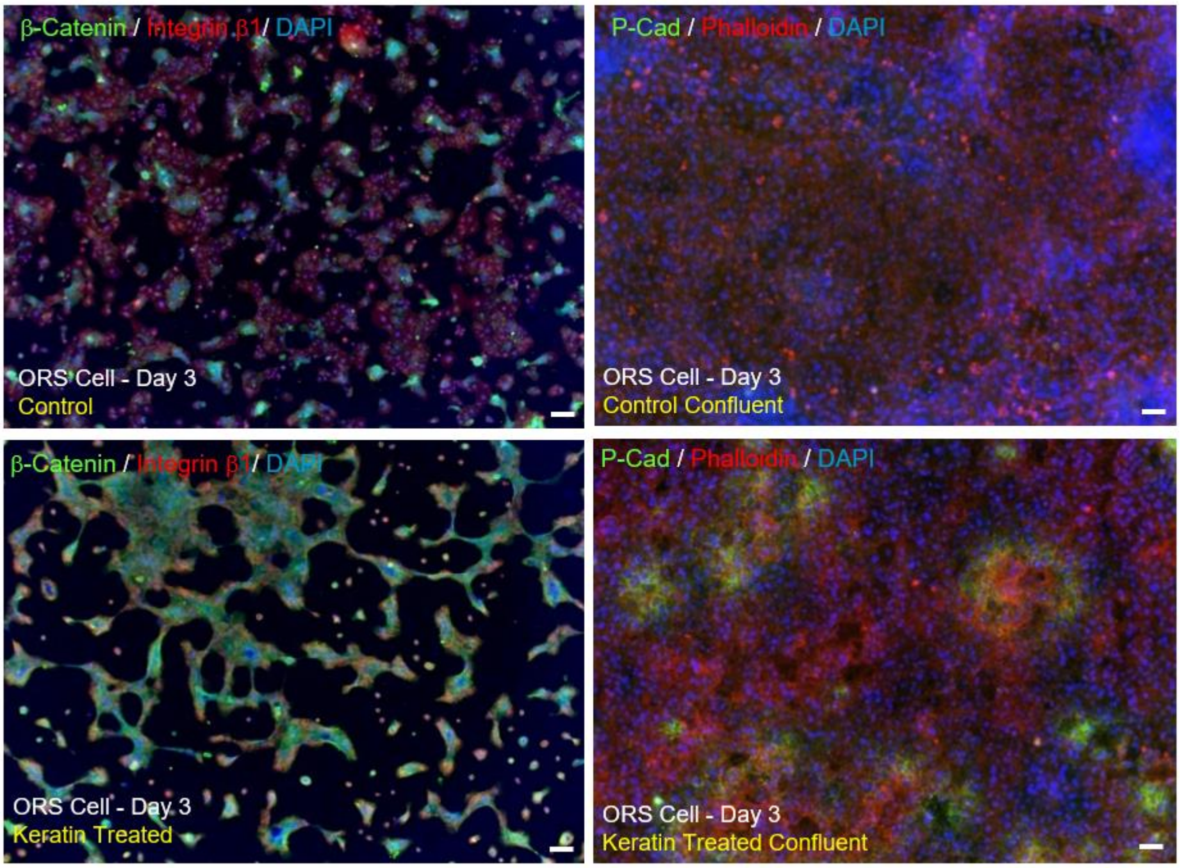

Supplementary Figure 15 | Hair-derived keratin induced P-cadherin expressing germ formation of ORS cells. *In vitro* germ formation of ORS cells by immunofluorescent staining of in the presence of keratin (low magnified images); DAPI, blue; phalloidin, integrin β1, red; P-cadherin, β-catenin, green; Control confluent, ORS cell culture at confluent cell density under ORS culture medium; Keratin treated confluent, ORS cell culture at confluent cell density in the presence of keratin. Scale bars, 100μm.

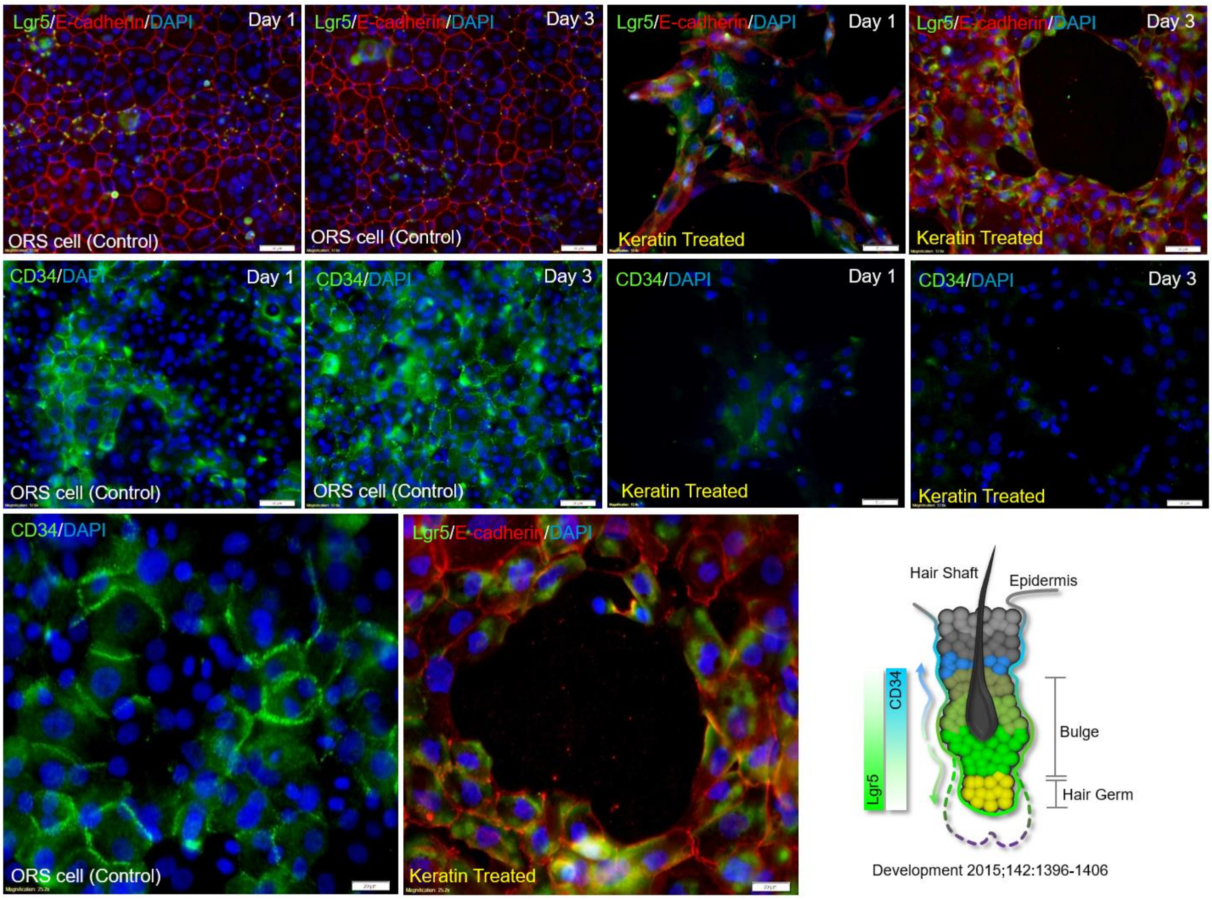

Supplementary Figure 16 | Hair-derived keratin induced the generation of Lgr5-positive cell population from ORS cells. Images of CD34 and Lgr5 expressions in ORS cells on day 1 and 3 after keratin treatment by immunofluorescent staining: DAPI, blue; E-cadherin, red; P-cadherin, CD34, Lgr5, green; Scale bars, 50μm and 20μm.

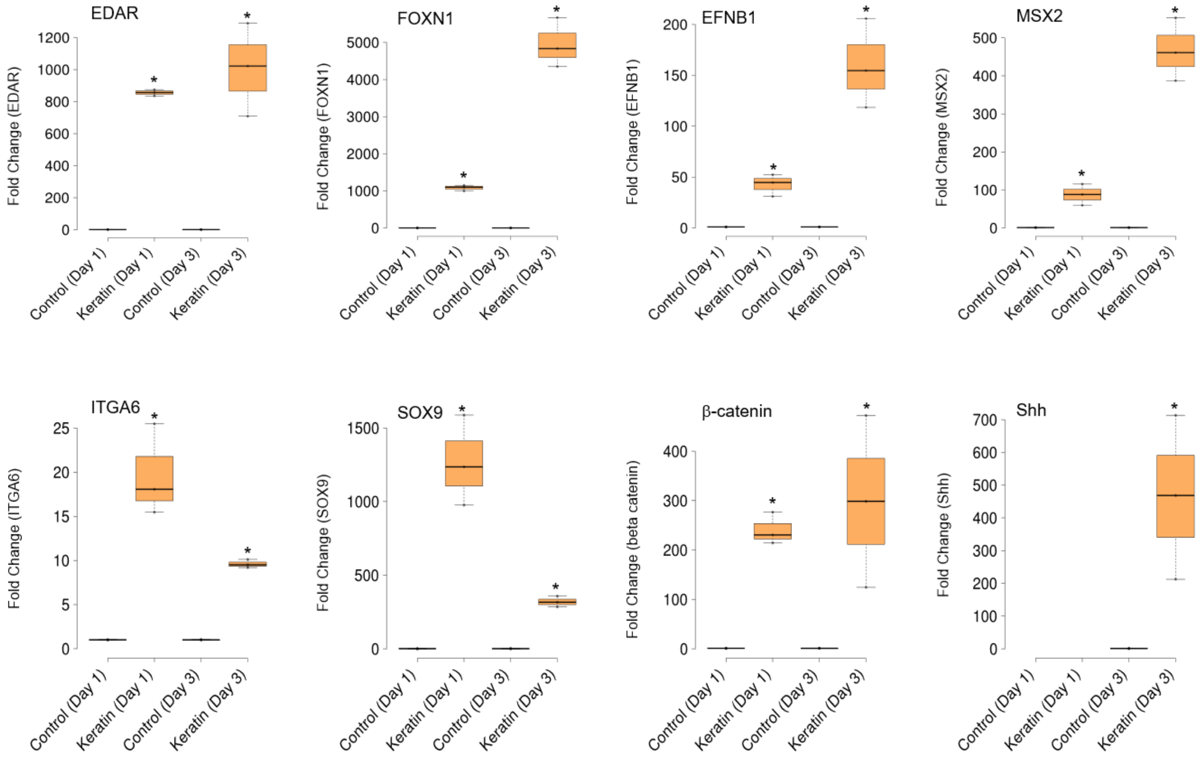

Supplementary Figure 17 | Hair keratin treatment upregulated ORS cell differentiation-related gene expressions. Graphical representation of realtime-qPCR analysis for *EDAR*, *FOXN1*, *EFNB1*, *MSX2*, *ITGA6*, *SOX9*, *β-catenin* and *SHH* mRNA expressions in ORS cell culture on day 1 and 3 after keratin treatment. *P,0.01, indicates a difference between control and keratin treated. (n=4; mean ± standard deviation (s.d.)).

a

Ensembl ID	Gene symbol	OC_GC	OK_GC	FC_OK/OC
ENSG00000167768	KRT1	36	15	0.405113
ENSG00000172867	KRT2	0	0	
ENSG00000186442	KRT3	15	20	1.29636
ENSG00000170477	KRT4	10	151	14.68128
ENSG00000186081	KRT5	340838	301806	0.860928
ENSG00000205420	KRT6A	102775	115064	1.088526
ENSG00000185479	KRT6B	38756	62153	1.55923
ENSG00000170465	KRT6C	1850	1250	0.656939
ENSG00000135480	KRT7	20079	14731	0.713308
ENSG00000170421	KRT8	3178	4522	1.383451
ENSG00000171403	KRT9	81	50	0.600167
ENSG00000186395	KRT10	568	518	0.886683
ENSG00000187242	KRT12	NOT EXPRESSED		
ENSG00000171401	KRT13	34	177	5.061524
ENSG00000186847	KRT14	115591	81360	0.684343
ENSG00000171346	KRT15	1727	8620	4.852906
ENSG00000186832	KRT16	10297	22796	2.152459
ENSG00000128422	KRT17	128368	163676	1.239696
ENSG00000111057	KRT18	1756	2999	1.6605
ENSG00000171345	KRT19	586	4614	7.655384
ENSG00000171431	KRT20	0	0	
ENSG00000108244	KRT23	86	1987	22.46396
ENSG00000167916	KRT24	2	2	0.97227
ENSG00000204897	KRT25	NOT EXPRESSED		
ENSG00000186393	KRT26	NOT EXPRESSED		
ENSG00000171446	KRT27	0	1	
ENSG00000173908	KRT28	NOT EXPRESSED		

Ensembl ID	Gene symbol	OC_GC	OK_GC	FC_OK/OC
ENSG00000094796	KRT31	98	149	1.478248
ENSG00000108759	KRT32	NOT EXPRESSED		
ENSG00000006059	KRT33A	1	6	5.833621
ENSG00000131738	KRT33B	22	72	3.181975
ENSG00000131737	KRT34	727	3663	4.898798
ENSG00000197079	KRT35	0	1	
ENSG00000126337	KRT36	0	2	
ENSG00000108417	KRT37	4	46	11.18111
ENSG00000171360	KRT38	0	3	
ENSG00000196859	KRT39	NOT EXPRESSED		
ENSG00000204889	KRT40	0	1	
ENSG00000139648	KRT71	2	0	
ENSG00000170486	KRT72	0	0	
ENSG00000186049	KRT73	2	2	0.97227
ENSG00000170484	KRT74	2	3	1.458405
ENSG00000170454	KRT75	3576	4873	1.324909
ENSG00000185069	KRT76	0	1	
ENSG00000189182	KRT77	1	1	0.97227
ENSG00000170423	KRT78	49	246	4.881193
ENSG00000185640	KRT79	13	79	5.908411
ENSG00000167767	KRT80	387	4411	11.08187
ENSG00000205426	KRT81	0	1	
ENSG00000161850	KRT82	1	2	1.94454
ENSG00000170523	KRT83	NOT EXPRESSED		
ENSG00000161849	KRT84	2	3	1.458405
ENSG00000135443	KRT85	NOT EXPRESSED		
ENSG00000170442	KRT86	9	12	1.29636

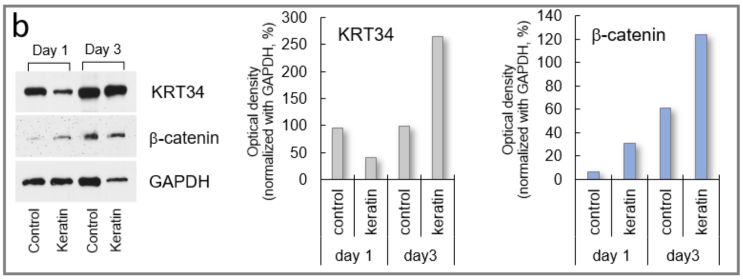

Supplementary Figure 18 | Gene expression profiles of keratin family genes in ORS cells in the absence and presence of keratin. a: RNA-Seq method had been used to measure the gene expression levels of keratin genes. OC_GC: the number of raw sequencing read mapped on the keratin gene in the ORS cell in the absence of keratin. OK_GC: the number of raw sequencing read mapped on the keratin gene in the ORS cell in the presence of keratin. FC: fold change between two samples using normalized expression value (RPKM, see the method section). b: Graphical representation of KRT34 and β-catenin molecular expressions of ORS cells in the presence of keratin: western blot assay.

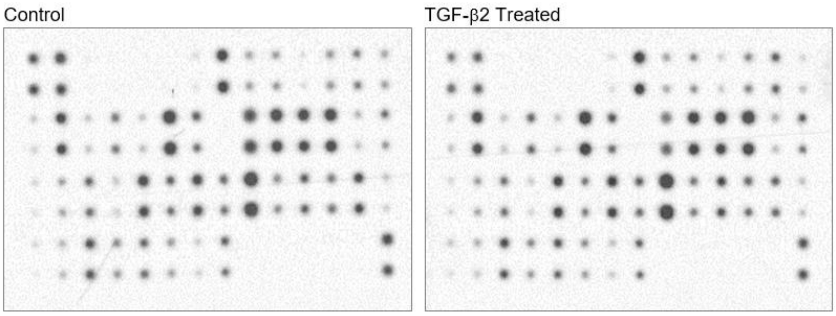

	A	B	C	D	E	F	G	H	I	J	K	L	M	N
1	Pos	Pos	Neg	Neg	Blank	Blank	Bad	Bax	Bcl-2	Bcl-w	BID	BIM	Caspase-3	Caspase-8
2	Pos	Pos	Neg	Neg	Blank	Blank	Bad	Bax	Bcl-2	Bcl-w	BID	BIM	Caspase-3	Caspase-8
3	CD40	CD40L	cIAP-2	cytoC	DR6	Fas	FasL	Blank	HSP27	HSP60	HSP70	HTRA	IGF-I	IGF-II
4	CD40	CD40L	cIAP-2	cytoC	DR6	Fas	FasL	Blank	HSP27	HSP60	HSP70	HTRA	IGF-I	IGF-II
5	IGFBP-1	IGFBP-2	IGFBP-3	IGFBP-4	IGFBP-5	IGFBP-6	IGF-1sR	Livin	p21	p27	p53	SMAC	Survivin	sTNF-R1
6	IGFBP-1	IGFBP-2	IGFBP-3	IGFBP-4	IGFBP-5	IGFBP-6	IGF-1sR	Livin	p21	p27	p53	SMAC	Survivin	sTNF-R1
7	sTNF-R2	TNF-α	TNF-β	TRAILR-1	TRAILR-2	TRAILR-3	TRAILR-4	XIAP	Blank	Blank	Neg	Neg	Neg	Pos
8	sTNF-R2	TNF-α	TNF-β	TRAILR-1	TRAILR-2	TRAILR-3	TRAILR-4	XIAP	Blank	Blank	Neg	Neg	Neg	Pos

Supplementary Figure 19 | Images of apoptosis array of ORS cells and TGF-β2 treated ORS cells.

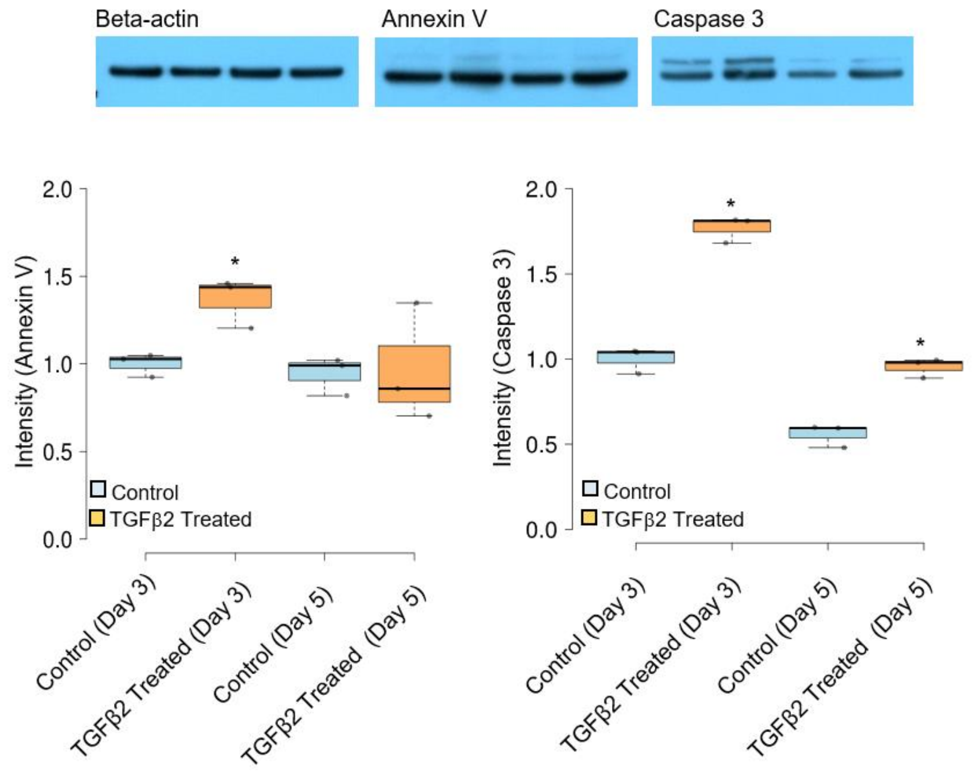

Supplementary Figure 20 | Western blot analysis of ORS cells and TGF-β2 treated ORS cells. Graphical representation of annexin V and caspase 3 molecular expressions in ORS cell culture on day 3 and 5 after TGF-β2 treatment. *P,0.01, indicates a difference between control and TGF-β2 treated. (n=3; mean ± standard deviation (s.d.)).

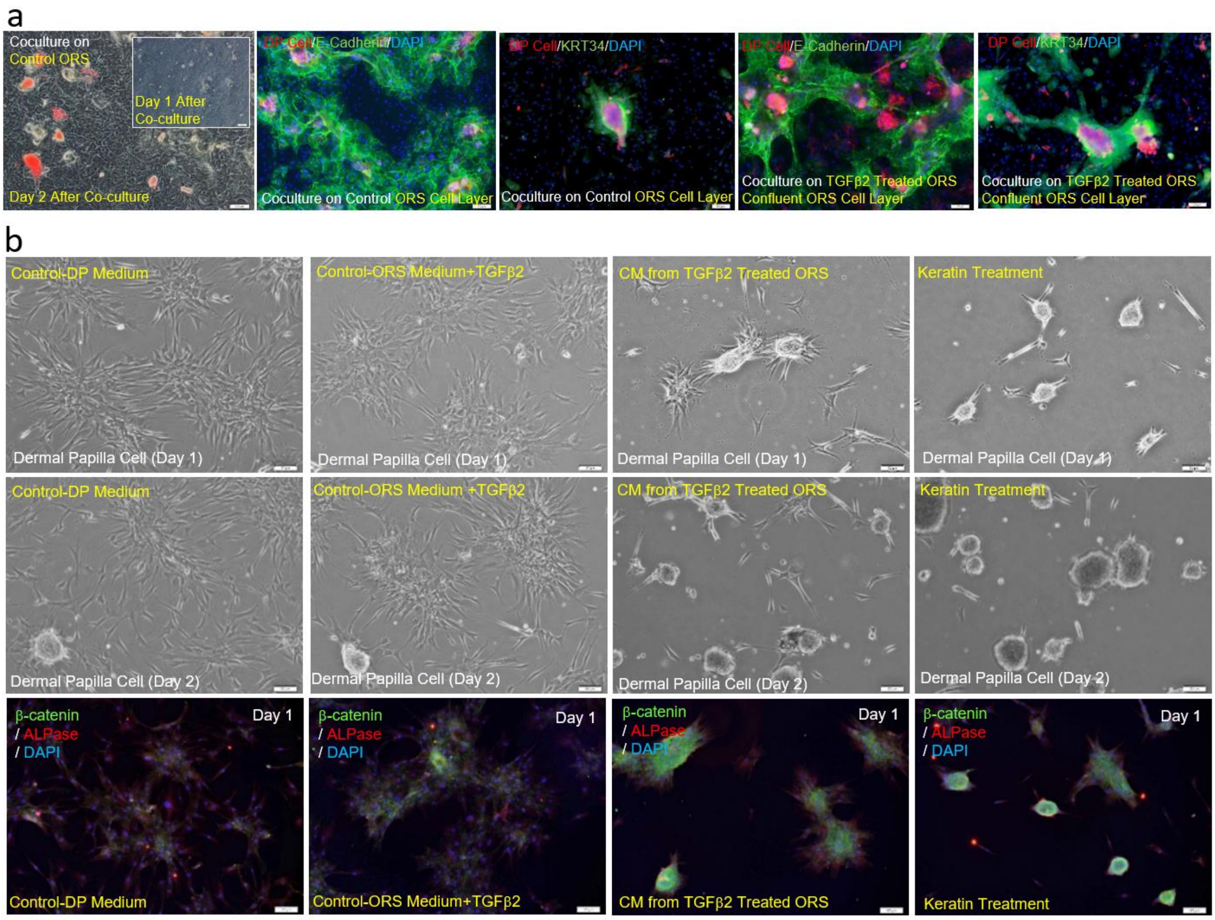

Supplementary Figure 21 | DP cell condensation in co-culture with TGFβ2-treated ORS cells and in conditioned medium collected from TGFβ2-treated ORS cell culture. a: Images of DP cell condensation on TGFβ2-treated ORS cell layers. Co-culture of cell tracker-treated DP cells (red) with TGFβ2-treated ORS cell layers. Immunofluorescent image; cell tracker-treated DP cells, red; E-cadherin, KRT34, green; DAPI, blue. Scale bars, 100μm. b: Images of DP cell condensation in conditioned medium collected from TGFβ2-treated ORS cell culture, and DP cell condensation by immunofluorescent staining; alkaline phosphatase, red; β-catenin, green; DAPI, blue; Control-DP medium, DP culture medium; Control-ORS Medium-TGFβ2, basic ORS medium including TGFβ2; CM from TGFβ2 treated ORS, conditioned medium collected from TGFβ2-treated ORS cell culture; Keratin treatment, DP medium containing 1(w/v)% keratin. Scale bars, 100μm.

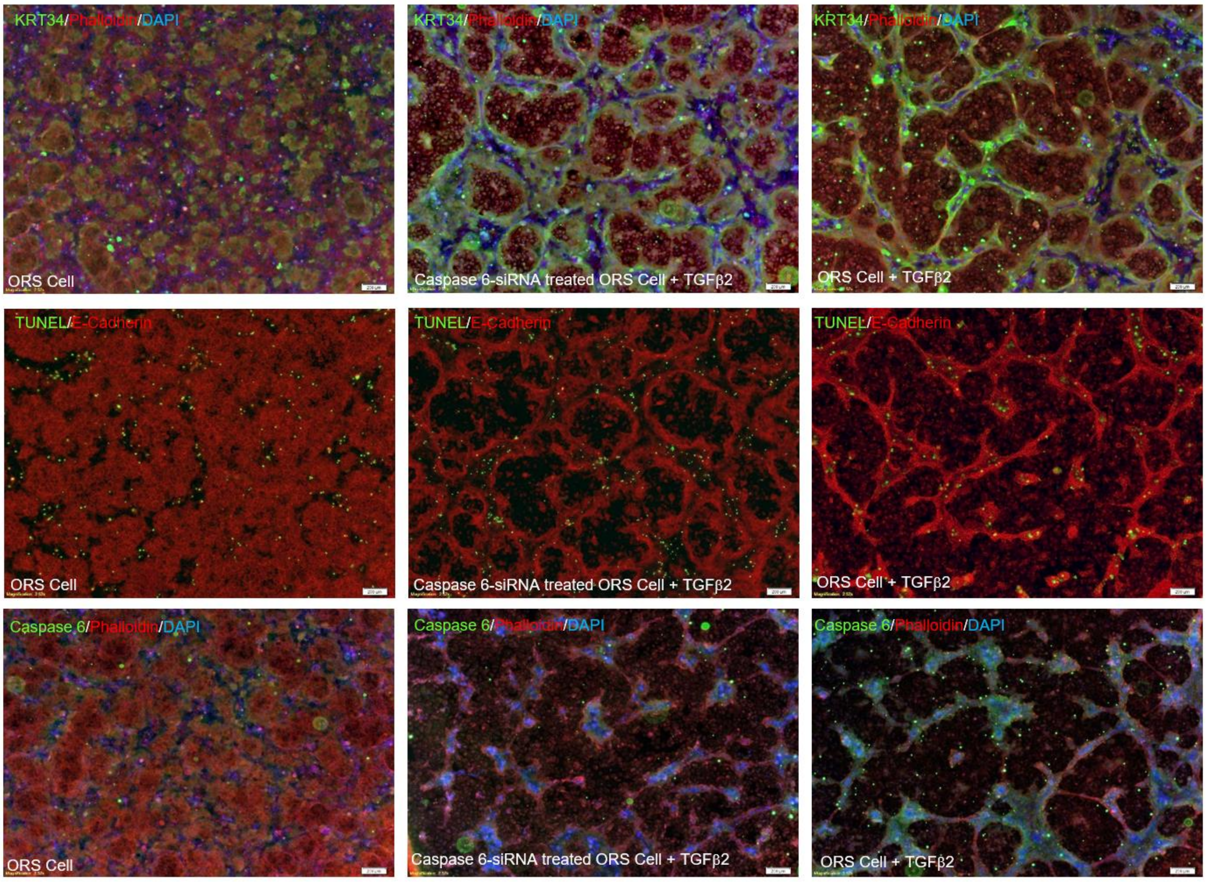

Supplementary Figure 22 | Low magnified images of ORS cells, ORS cell culture in the presence of TGFβ2 and caspase 6-silenced ORS cell culture in the presence of TGFβ2 by immunofluorescent staining; Phalloidin, E-cadherin, red; KRT34, tunel, caspase 6, green; DAPI, blue. Scale bars, 200μm.

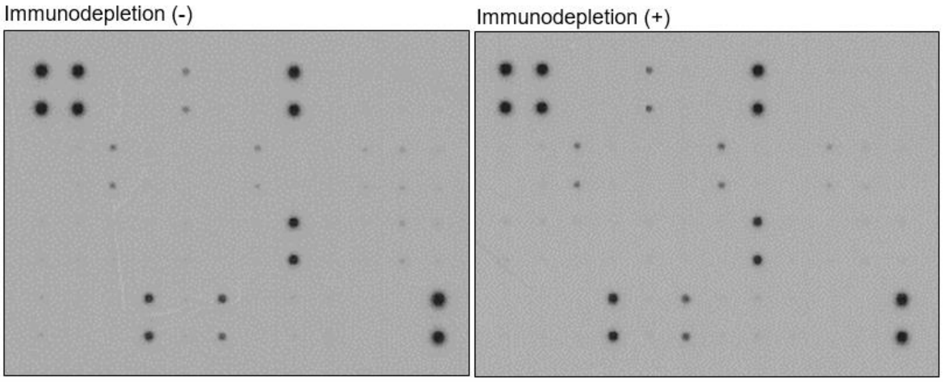

	A	B	C	D	E	F	G	H	I	J	K	L
1	Pos	Pos	Neg	Neg	AR	bFGF	bNGF	EGF	EGF R	FGF-4	FGF-6	FGF-7
2	Pos	Pos	Neg	Neg	AR	bFGF	bNGF	EGF	EGF R	FGF-4	FGF-6	FGF-7
3	GCSF	GDNF	GM-CSF	HB-EGF	HGF	IGFBP-1	IGFBP-2	IGFBP-3	IGFBP-4	IGFBP-6	IGF-I	IGF-I SR
4	GCSF	GDNF	GM-CSF	HB-EGF	HGF	IGFBP-1	IGFBP-2	IGFBP-3	IGFBP-4	IGFBP-6	IGF-I	IGF-I SR
5	IGF-II	M-CSF	M-CSF R	NT-3	NT-4	PDGF Rα	PDGF Rβ	PDGF-AA	PDGF-AB	PDGF-BB	PIGF	SCF
6	IGF-II	M-CSF	M-CSF R	NT-3	NT-4	PDGF Rα	PDGF Rβ	PDGF-AA	PDGF-AB	PDGF-BB	PIGF	SCF
7	SCF R	TGF-α	TGF-β	TGF-β2	TGF-β3	VEGF	VEGF R2	VEGF R3	VEGF–D	BLANK	BLANK	Pos
8	SCF R	TGF-α	TGF-β	TGF-β2	TGF-β3	VEGF	VEGF R2	VEGF R3	VEGF-D	BLANK	BLANK	Pos

Supplementary Figure 23 | Images of growth factor array of the conditioned medium and the keratin-eliminated conditioned medium collected from TGFβ2-treated ORS cell culture.

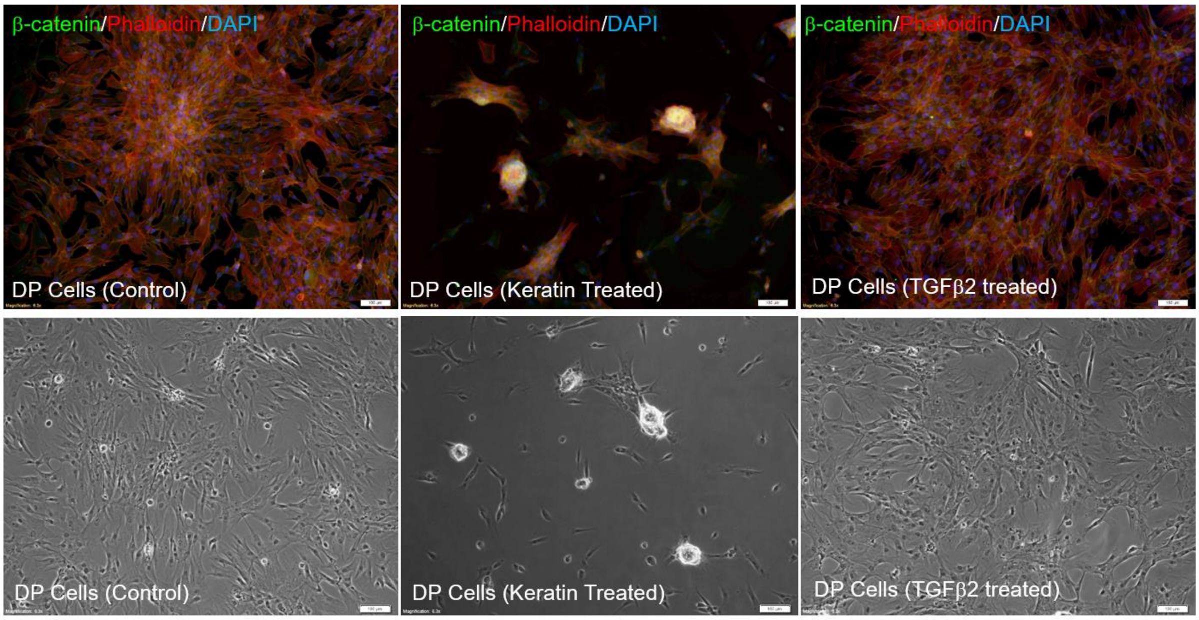

Supplementary Figure 24 | TGFβ2 did not influence DP cell condensation by itself. Images of DP cells in the presence of TGFβ2 or not by immunofluorescent staining; Phalloidin, red; β-catenin, green; DAPI, blue. Scale bars, 100μm.

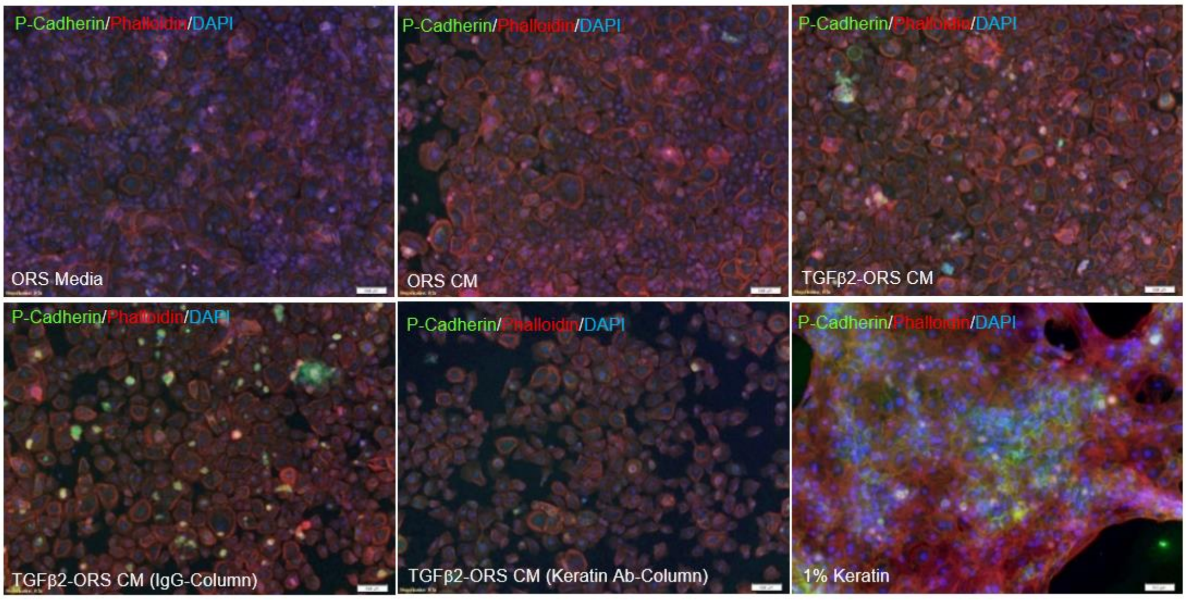

Supplementary Figure 25 | The released keratin in conditioned medium from TGFβ2-treated ORS cell culture was not efficient to induce P-cadherin expressing germ formation. Germ formation of ORS cells in conditioned medium from TGFβ2-treated ORS cell culture and keratin-eliminated conditioned medium of TGFβ2-treated ORS cell culture using a column containing anti-human hair keratin antibody-conjugated beads by immunofluorescent staining; DAPI, blue; phalloidin, red; P-cadherin, green: ORS media, ORS culture medium; ORS CM, conditioned medium from ORS cell culture; TGFβ2-ORS CM, conditioned medium collected from TGFβ2-treated ORS cell culture; TGFβ2-ORS CM (IgG-column), conditioned medium collected from TGFβ2-treated ORS cell culture and then treated with normal IgG-conjugated beads; TGFβ2-ORS CM (keratin Ab-column), keratin-eliminated conditioned medium collected from TGFβ2-treated ORS cell culture; 1% Keratin, DP medium containing 1(w/v)% keratin. Scale bars, 100μm.

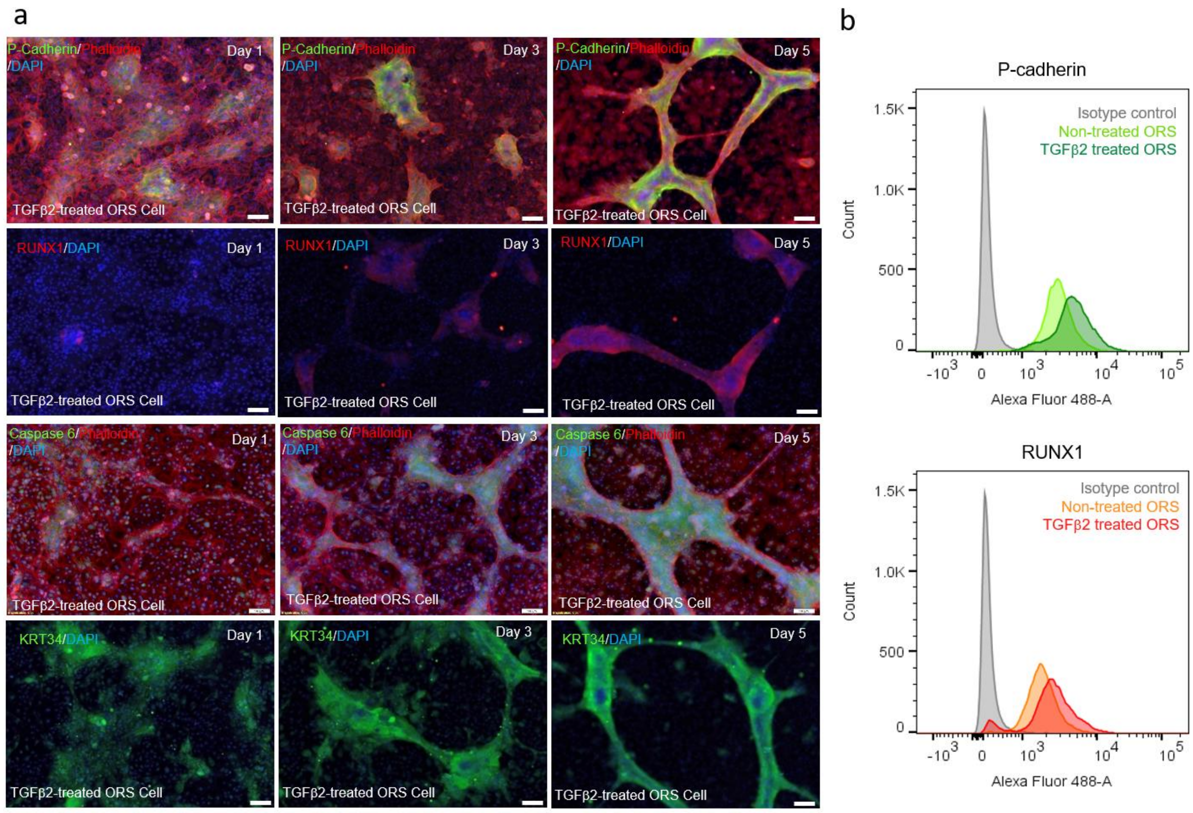

Supplementary Figure 26 | P-cadherin expressing germ formation of ORS cells in the presence of TGFβ2. a: Images of time-course P-cadherin expressing germ formation of ORS cells in the presence of TGFβ2 by immunofluorescent staining; DAPI, blue; phalloidin, RUNX1, red; P-cadherin, caspase 6, KRT34, green. Scale bars, 100 μm. b: Flow cytometric analysis of P-cadherin and RUNX1 of TGFβ2-treated ORS cells.

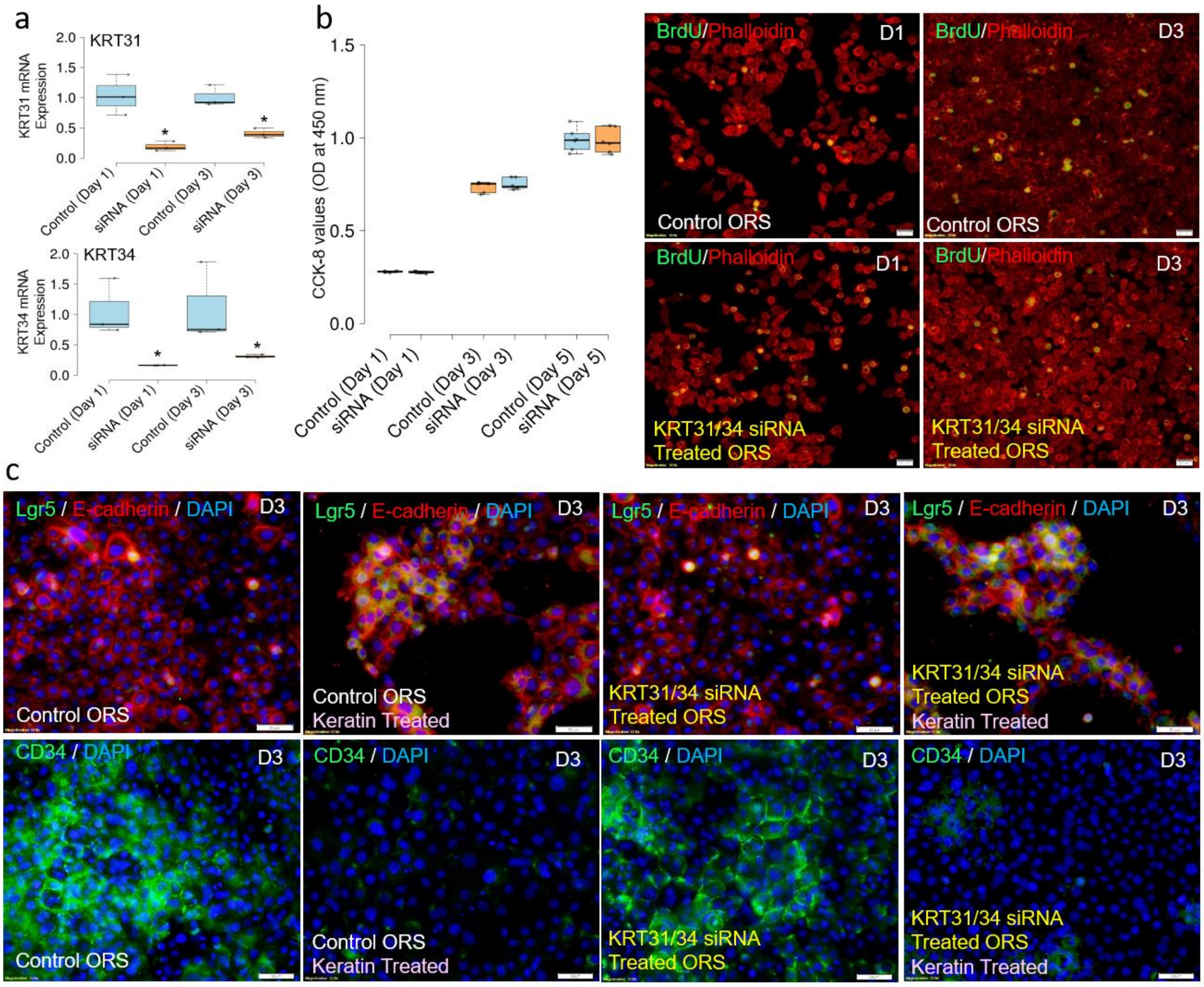

Supplementary Figure 27 | Silencing of KRT31/34 mRNA expression does not influence ORS growth and differentiation. a: Graphical representation of KRT31 and KRT34 mRNA expressions in control ORS cells transfected with negative control siRNA-loaded lipofetamine and KRT31/KRT34 silenced ORS cells on day 1 and 3 of culture. *P,0.01, indicates a difference between control and silenced ORS cells. (n=4; mean ± standard deviation (s.d.)). b: Quantification of cell growth of control ORS cells transfected with negative control siRNA-loaded lipofetamine and KRT31/KRT34 silenced ORS cells. *P,0.01, indicates a difference between control and silenced ORS cells. (n=4; mean ± standard deviation (s.d.)). Image of ORS cells on day 1 and 3 of culture by immunofluorescent staining; DAPI, blue; BrdU, green; Phalloidin, red. Scale bars, 20μm. c. Immunocytochemial images of control ORS cells transfected with negative control siRNA-loaded lipofetamine and KRT31/KRT34 silenced ORS cells in the presence of keratin; E-cadherin, red; CD34, Lgr5, green; DAPI, blue. Scale bars, 50μm.

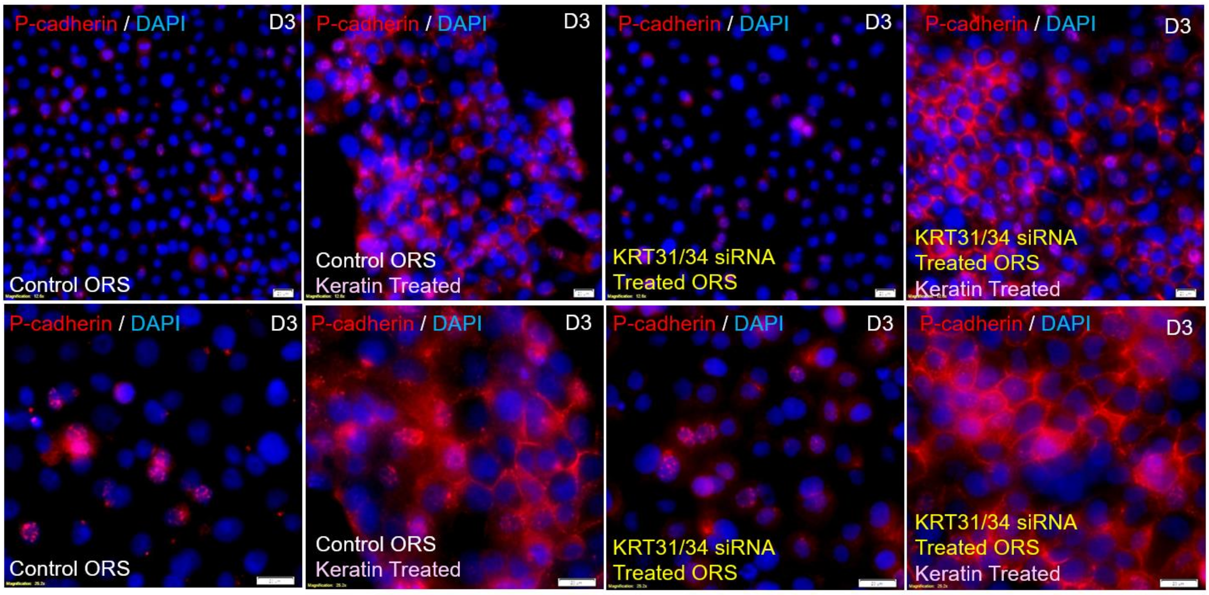

Supplementary Figure 28 | Immunocytochemial images of control ORS cells transfected with negative control siRNA-loaded lipofetamine and KRT31/KRT34 silenced ORS cells in the presence of keratin; P-cadherin, red; DAPI, blue. Scale bars, 20μm.

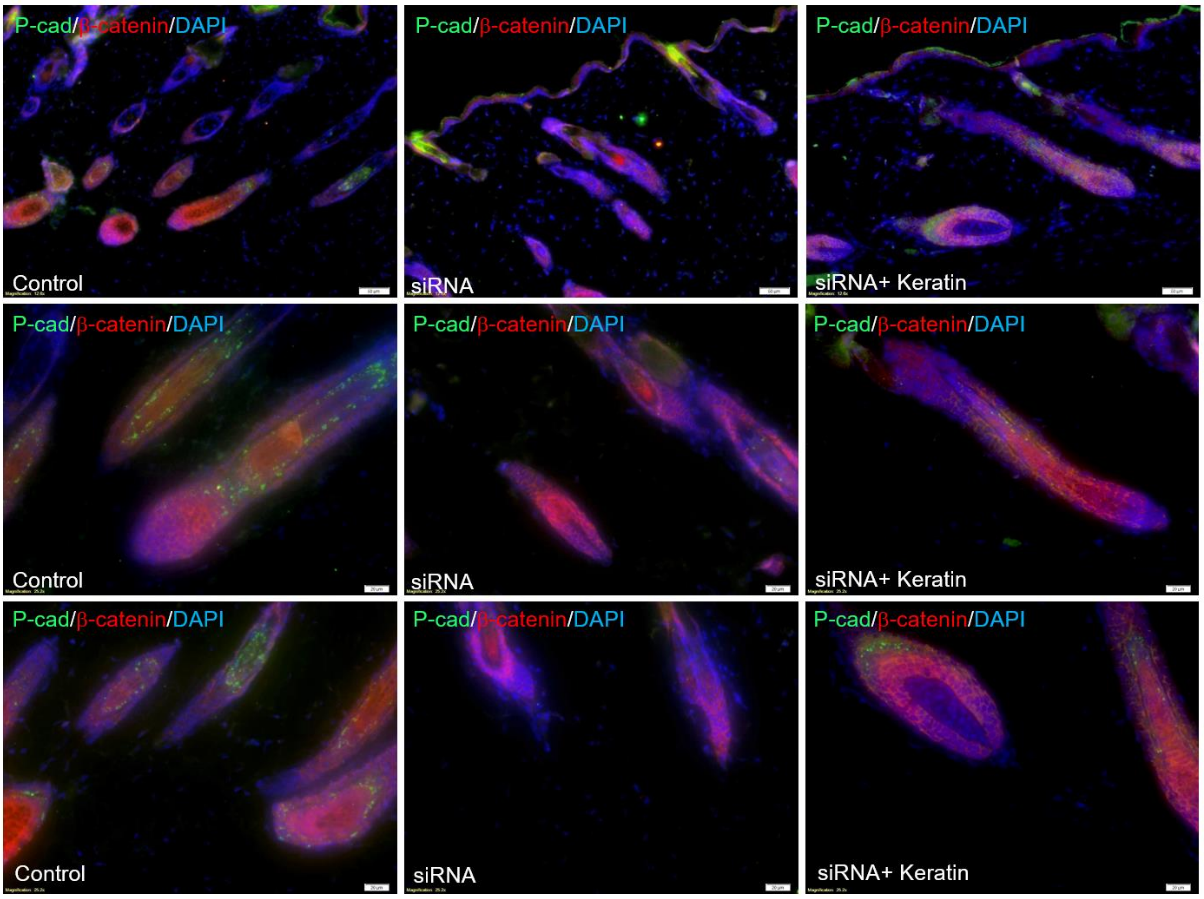

Supplementary Figure 29 | Immunohistochemical images of the back skin of mice on day 7 after KRT31/KRT34 siRNA transfection; Control, mice injected with negative control siRNA-loaded lipofetamine; siRNA, KRT31/KRT34 silenced mice; siRNA+Keratin, KRT31/KRT34 silenced and hair keratin injected mice; β-catenin, red; P-cadherin, green; DAPI, blue. Scale bars, 50μm and 20μm.

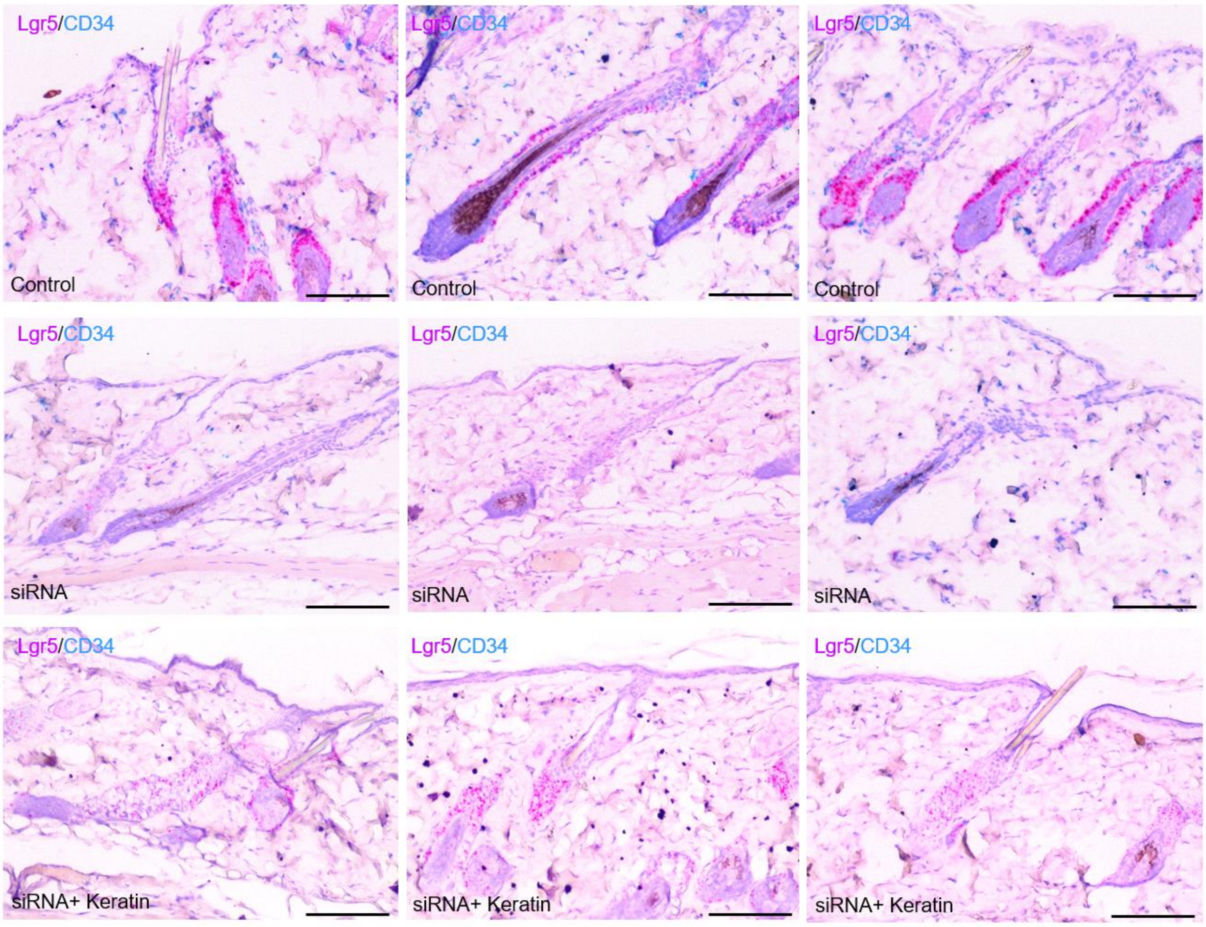

Supplementary Figure 30 | In situ hybridization analysis of the CD34 and Lgr5 mRNA of the back skin of mice on day 7 after KRT31/KRT34 siRNA transfection; Control, mice injected with negative control siRNA-loaded lipofetamine; siRNA, KRT31/KRT34 silenced mice; siRNA+Keratin, KRT31/KRT34 silenced and hair keratin injected mice; Lgr 5, red; CD34, blue. Scale bars, 100μm.

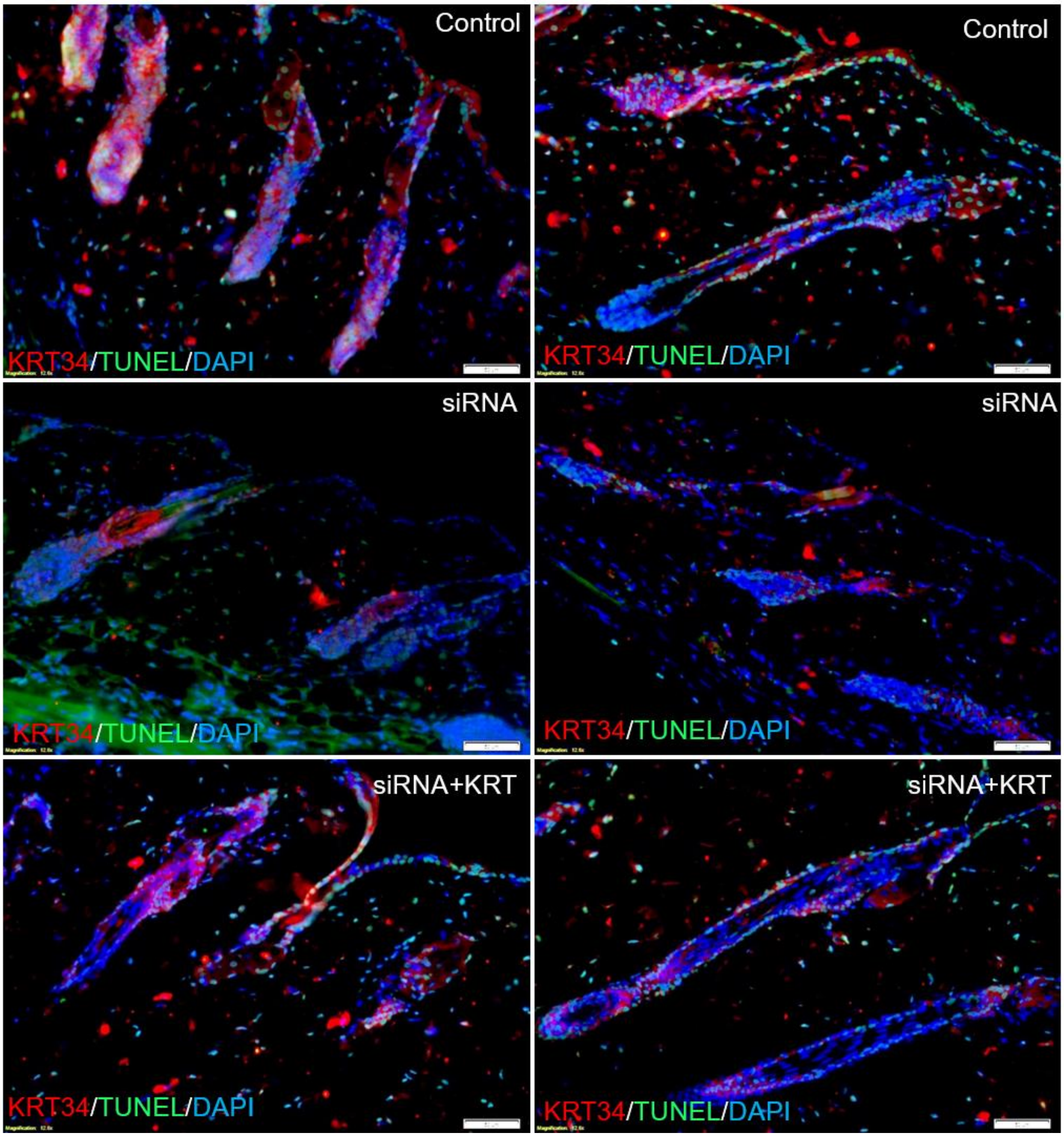

Supplementary Figure 31 | Immunohistochemical images of the back skin of mice on day 7 after KRT31/KRT34 siRNA transfection; Control, mice injected with negative control siRNA-loaded lipofetamine; siRNA, KRT31/KRT34 silenced mice; siRNA+KRT, KRT31/KRT34 silenced and hair keratin injected mice; KPT34, red; TUNEL, green; DAPI, blue. Scale bars, 50μm.

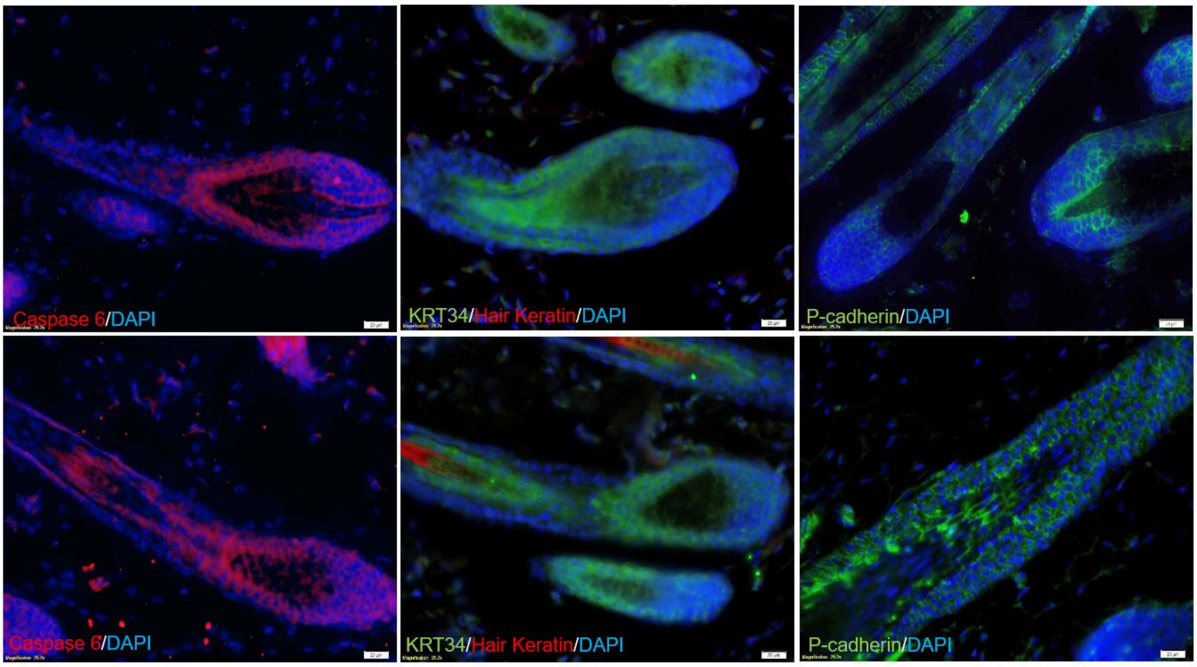

Supplementary Figure 32 | Immunohistochemical images of the back skin of control mice injected with negative control siRNA-loaded lipofetamine on day 7; caspase 6, type I+II hair keratin, red; P-cadherin, KRT34, green; DAPI, blue. Scale bars, 20μm.

Western Blot Analysis

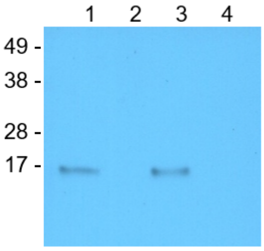

Lane 1 : rhFGF-20
Lane 2 : Hair keratin solution
Lane 3 : rhFGF-20 + Hair keratin solution
Lane 4 : FBS

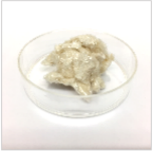

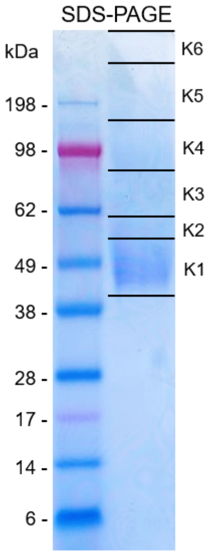

MALDI-TOF spectroscopy

[A]Band	Accession	[B]Score	[C]Matches	Description
K1	K1H1_HUMAN	239	2	Keratin, type I cuticular Ha1
	KT33B_HUMAN	239	2	Keratin, type I cuticular Ha3-II
K2	K1H1_HUMAN	251	2	Keratin, type I cuticular Ha1
	KT33B_HUMAN	251	2	Keratin, type I cuticular Ha3-II
	KRT81_HUMAN	115	2	Keratin, type II cuticular Hb1
	KRT83_HUMAN	115	2	Keratin, type II cuticular Hb3
	KRT85_HUMAN	115	2	Keratin, type II cuticular Hb5
	KRT86_HUMAN	115	2	Keratin, type II cuticular Hb6
K3	K1H1_HUMAN	198	1	Keratin, type I cuticular Ha1
	KT33B_HUMAN	198	1	Keratin, type I cuticular Ha3-II
K4	K1H1_HUMAN	159	1	Keratin, type I cuticular Ha1
	KT33B_HUMAN	159	1	Keratin, type I cuticular Ha3-II
	KRT83_HUMAN	82	2	Keratin, type II cuticular Hb3
	KRT85_HUMAN	82	2	Keratin, type II cuticular Hb5
	KRT86_HUMAN	82	2	Keratin, type II cuticular Hb6
K5	K1H1_HUMAN	165	1	Keratin, type I cuticular Ha1
	KT33B_HUMAN	165	1	Keratin, type I cuticular Ha3-II
K6	KRT83_HUMAN	94	2	Keratin, type II cuticular Hb3
	KRT85_HUMAN	94	2	Keratin, type II cuticular Hb5
	KRT86_HUMAN	94	2	Keratin, type II cuticular Hb6

Database search was limited to homo sapiens, and only results with a score of 50 or more were reported.
A : Results of analysis of gel bands from the following fractions.
B : Score is based on the scoring algorithm used by the MASCOT search engine using SwissProt as database. ($p<0.05$)
C : Matches indicates the number of peptides that match the protein.

Supplementary Figure 33 | Western blot analysis to identify the presence of FGF20, a potent inducer of DP condensation, in the extracted hair keratin and MALDI-TOF analysis to identify human hair-derived keratin.

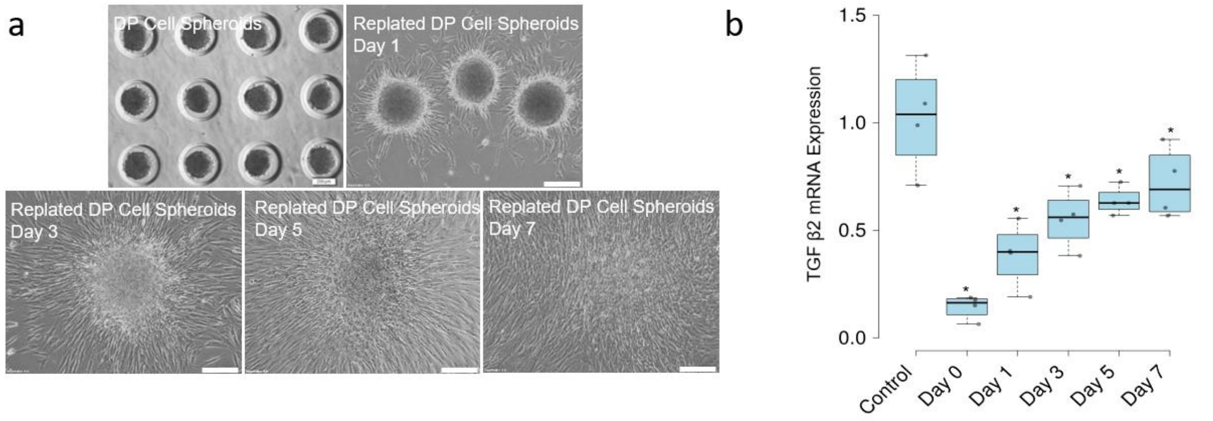

Supplementary Figure 34 | TGFβ2 expression was found to be downregulated during DP cell condensation and to be rapidly upregulated during the dispersion of condensed DP cells. a: Images of DP cell condensation via spontaneous self-aggregation within microwells and time-dependent dispersion of the replated DP cell spheroids. Scale bars, 200μm. b: Graphical quantification of TGFβ2 mRNA expressions according to the dispersion of condensed DP cells; real time-qPCR. *P,0.01, indicates a difference between control and replated DP cell spheroid culture; Control, DP cells; Day 0, DP cell spheroid within microwells; Day 1-7; replated DP cell spheroid culture on tissue culture plates for 1-7 days. (n=4; mean ± standard deviation (s.d.)).

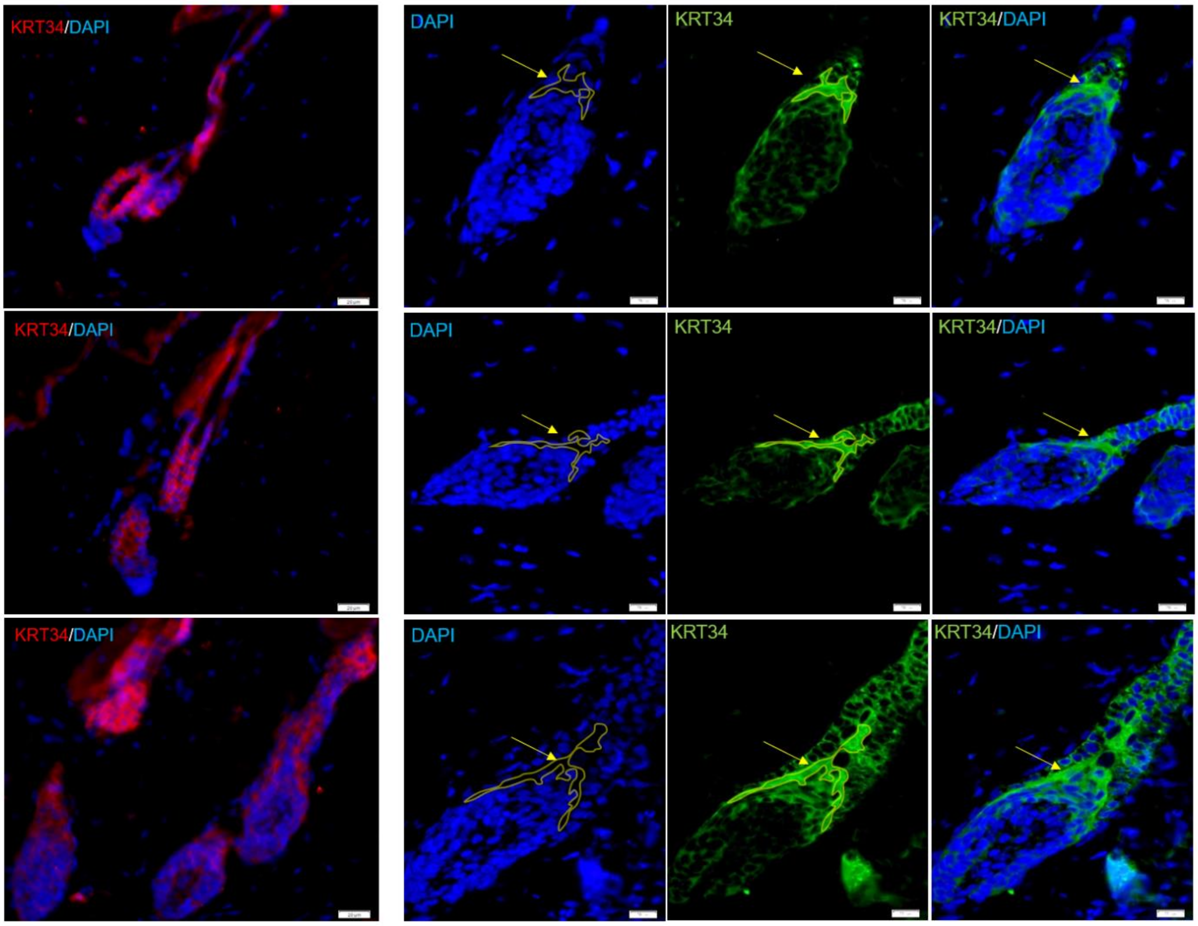

Supplementary Figure 35 | Keratin exposure from apoptotic cell death in developing hair follicles. Images of KRT34 exposure in hair follicles by immunohistological staining; DAPI, blue; KRT34, red or green. Scale bars, 20 μm. Yellow arrows indicate keratin exposure in void space unoccupied by cells

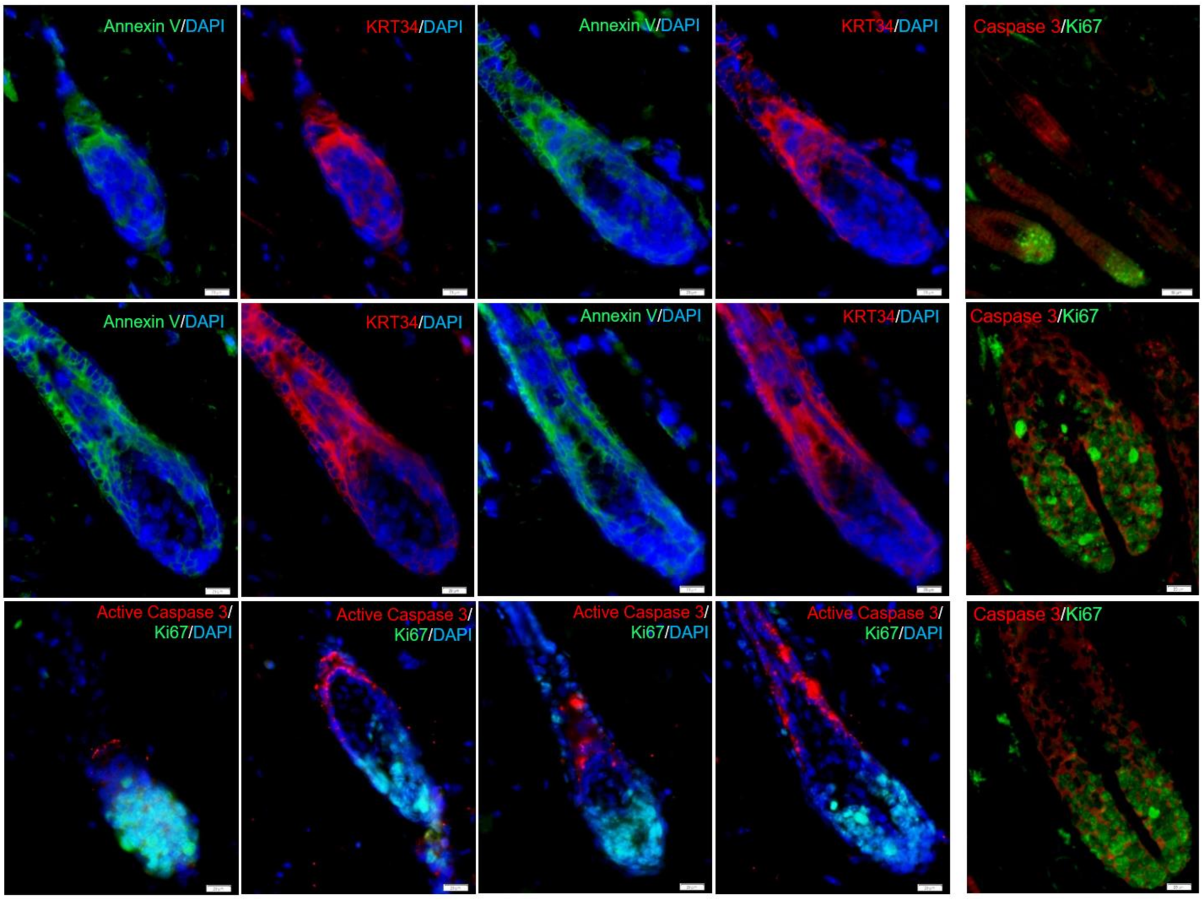

Supplementary Figure 36 | Proliferation and apoptosis in developing hair follicles of keratin-injected mice. Images of Annexin V, KRT34, caspase 3, active caspase 3 and Ki67 expression in hair follicles by immunohistological staining; DAPI, blue; KRT34, caspase 3, red; Annexin V, Ki67, green. Scale bars, 20 μm or 50 μm.

.

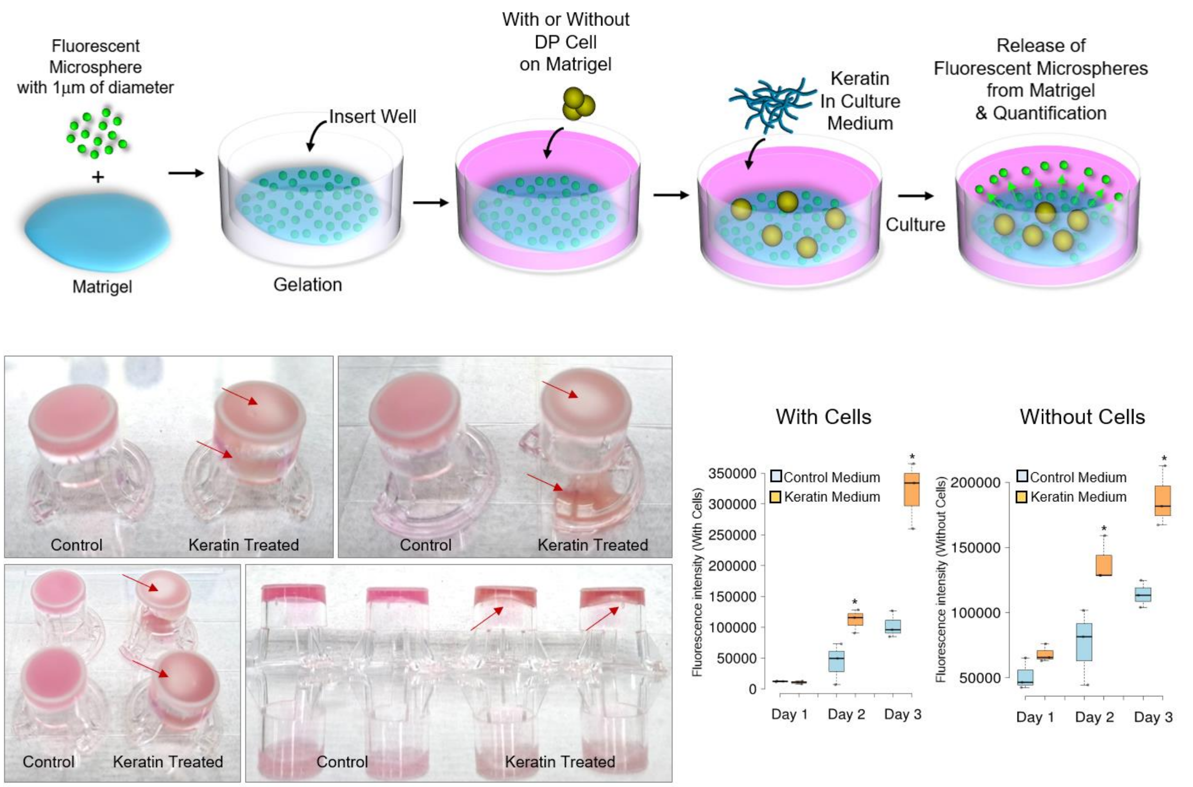

Supplementary Figure 37 | Hair keratin treatment resulted in partial degradation of Matrigel. Arrows indicate clear zone in Matrigel which resulted from partial degradation in the presence of keratin. Graphical quantification of fluorescent polystyrene microparticles in culture medium released from Matrigel on day 1, 2 and 3 after keratin treatment with DP cells or without DP cells. *P,0.01, indicates a difference between control and keratin treated; (n=4; mean ± standard deviation (s.d.)).

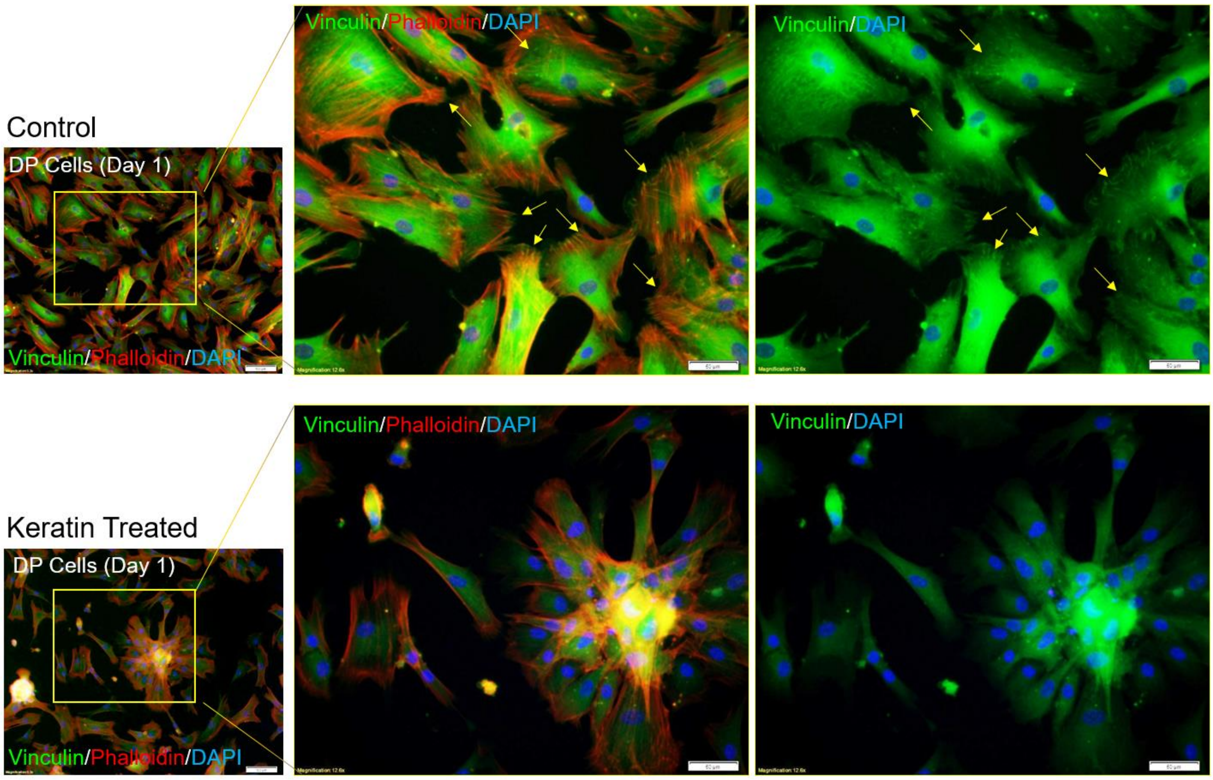

Supplementary Figure 38 | Hair keratin treatment induced the loss of vinculin expressions during DP cell condensation: Images of DP cell condensation on day 1 after keratin treatment by immunofluorescent staining; DAPI, blue; Phalloidin, red; vinculin, green. Scale bars, 50μm. Arrows indicate the expressed vinculin.

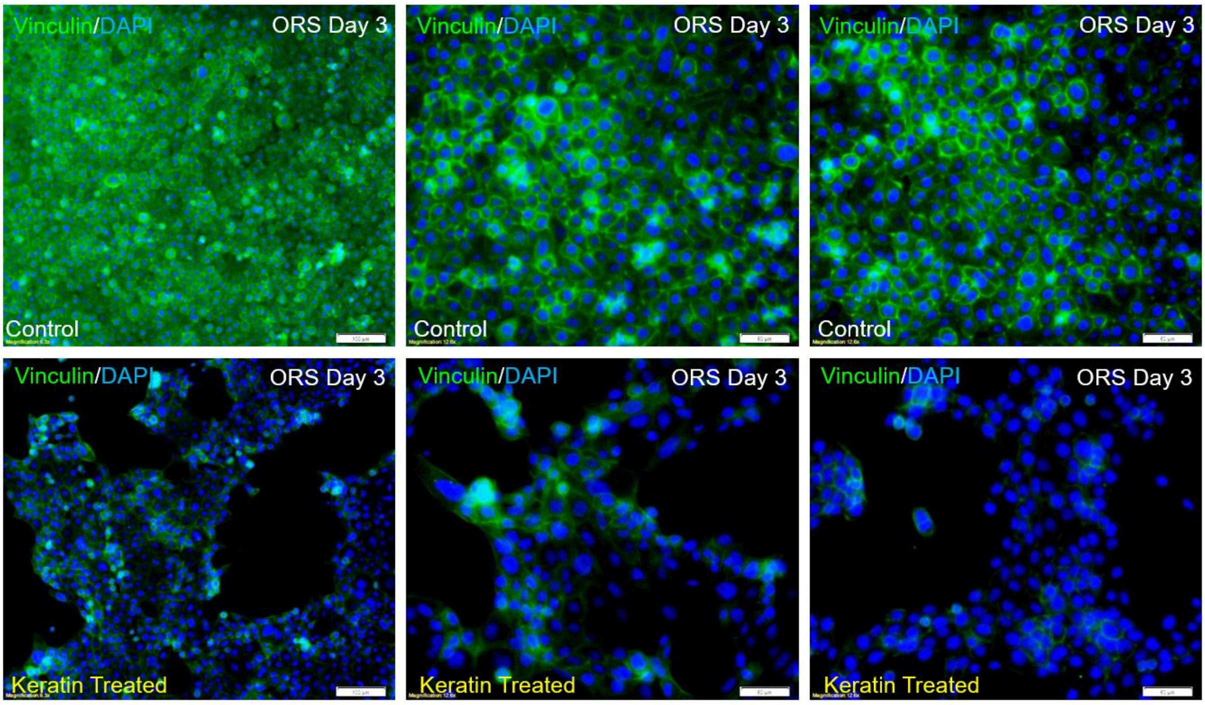

Supplementary Figure 39 | Hair keratin treatment induced the loss of vinculin expressions during ORS cells: Images of ORS cells on day 3 after keratin treatment by immunofluorescent staining; DAPI, blue; vinculin, green. Scale bars, 50µm.

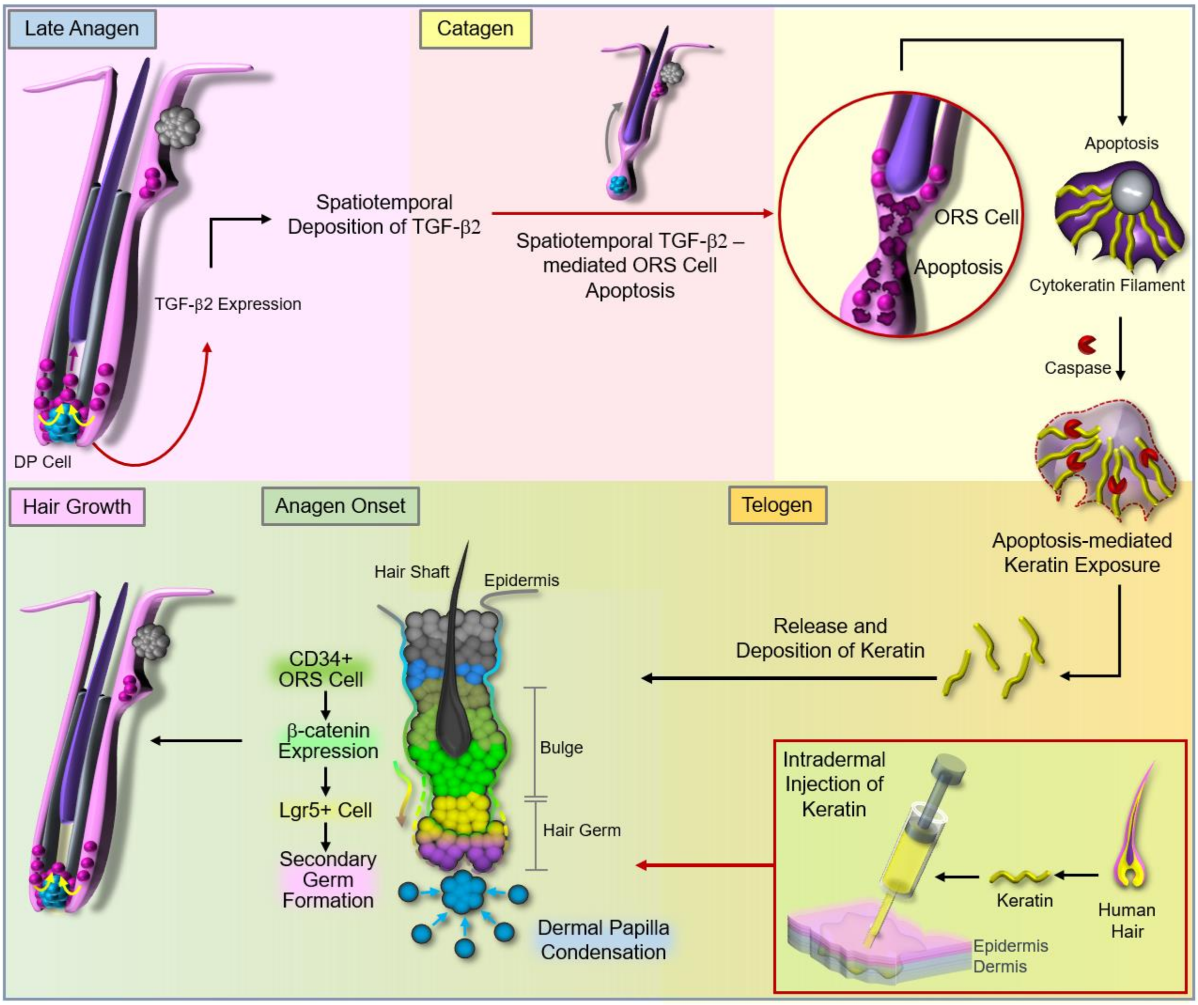

Supplementary Figure 40 | Schematic illustration of the mechanism of keratin-mediated hair growth

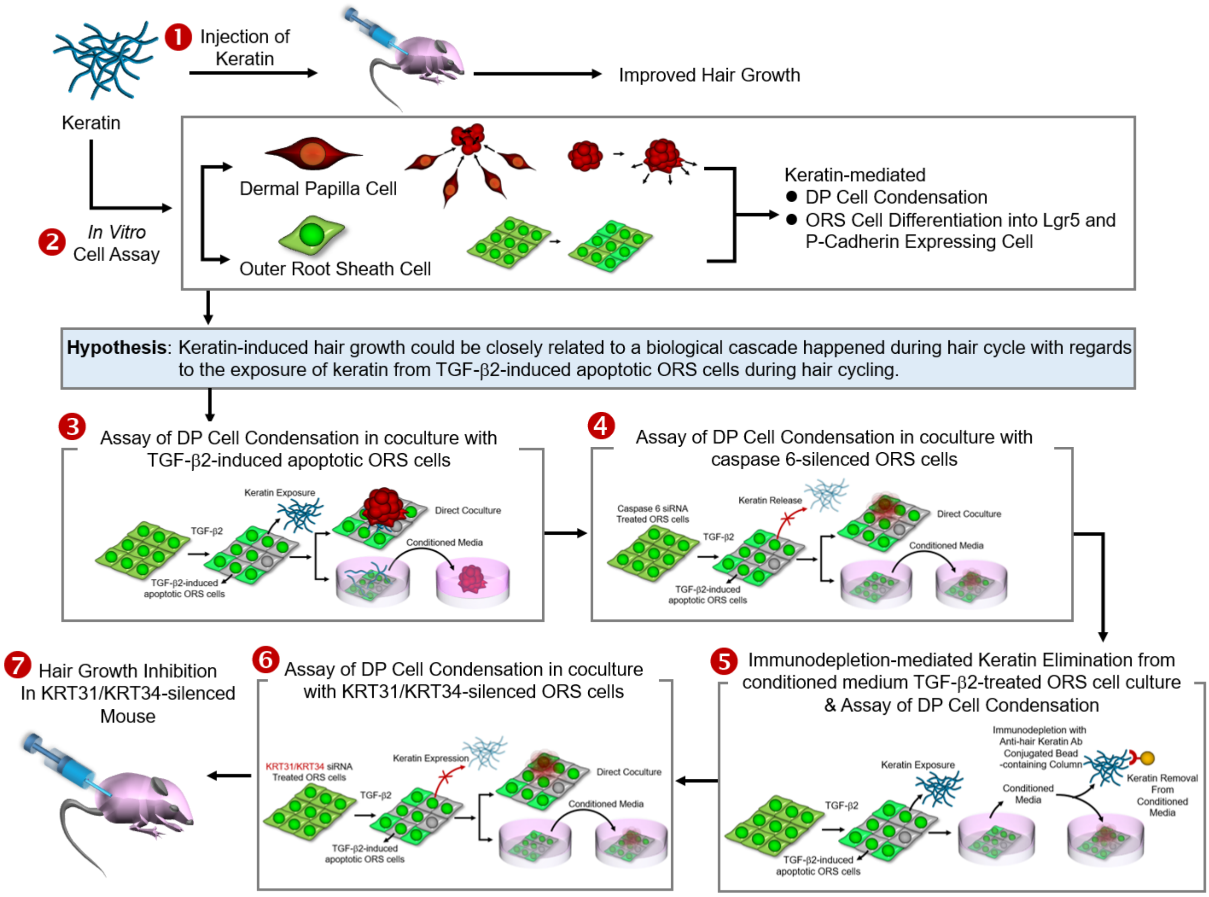

Supplementary Figure 41 | Schematic illustration of whole experimental procedure.

Supplementary Figure 42 | Full length uncropped original western blots and SDS-PAGE gels

Full length uncropped original western blot of Figure 4d

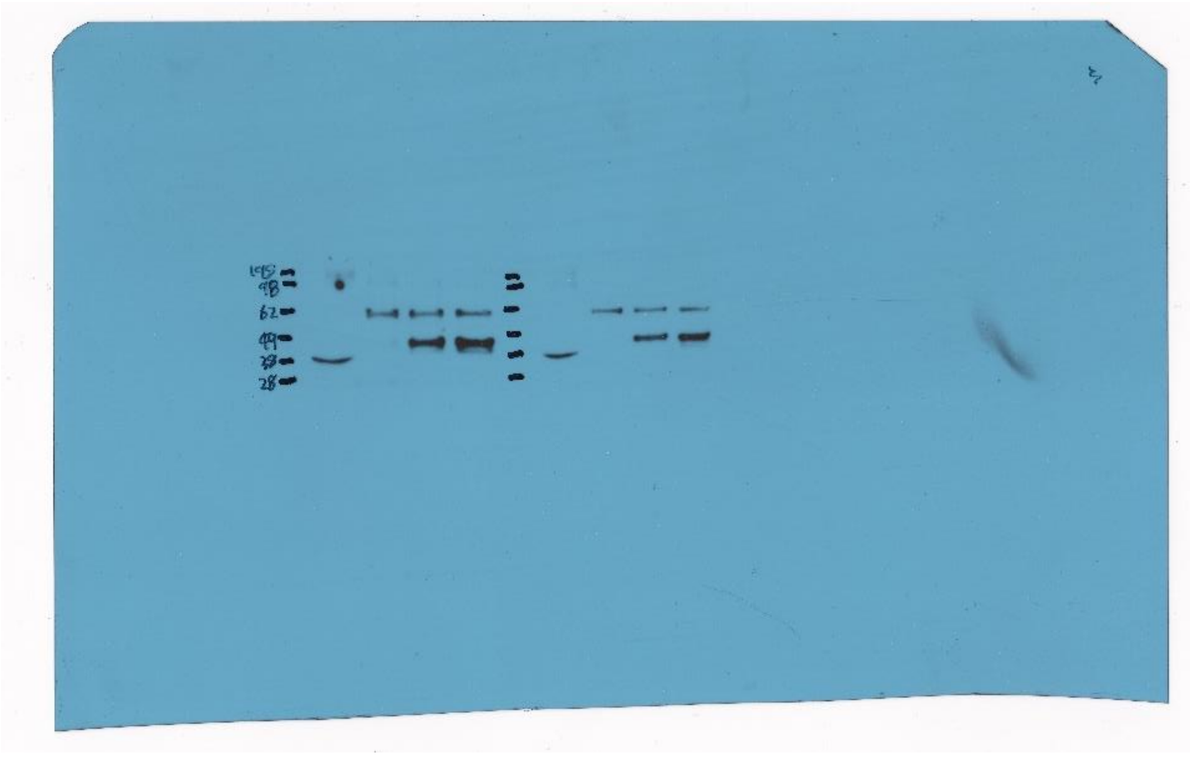

Full length uncropped original SDS-PAGE gels of Figure 5a

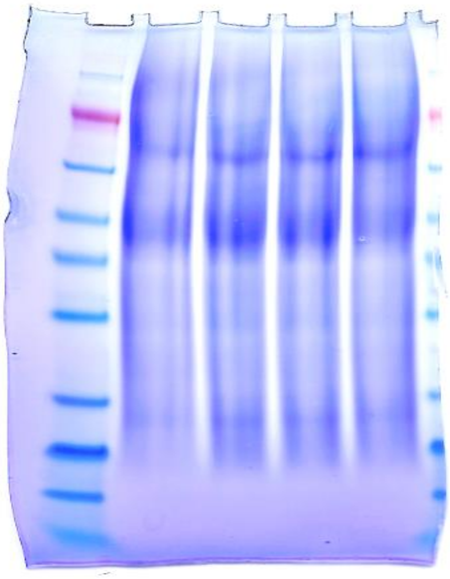

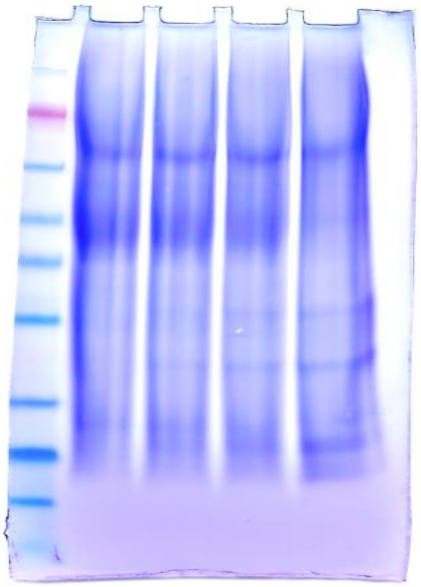

Full length uncropped original western blots of Figure 5c

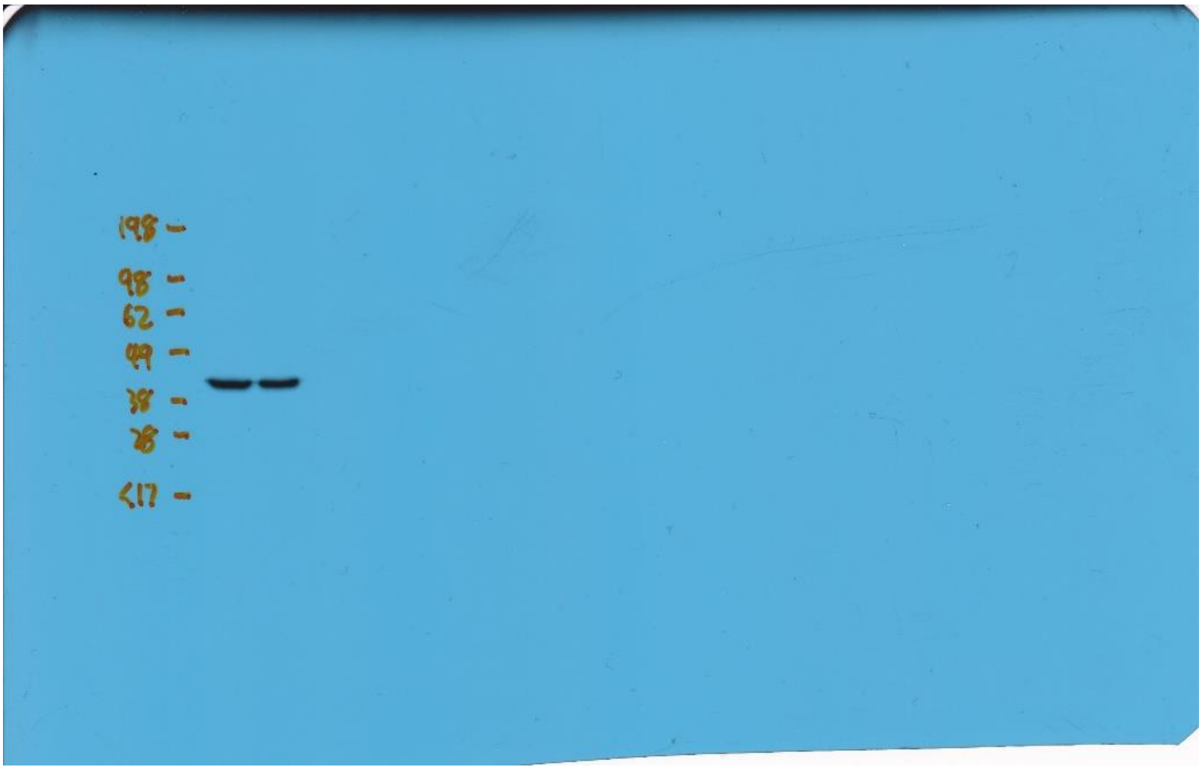

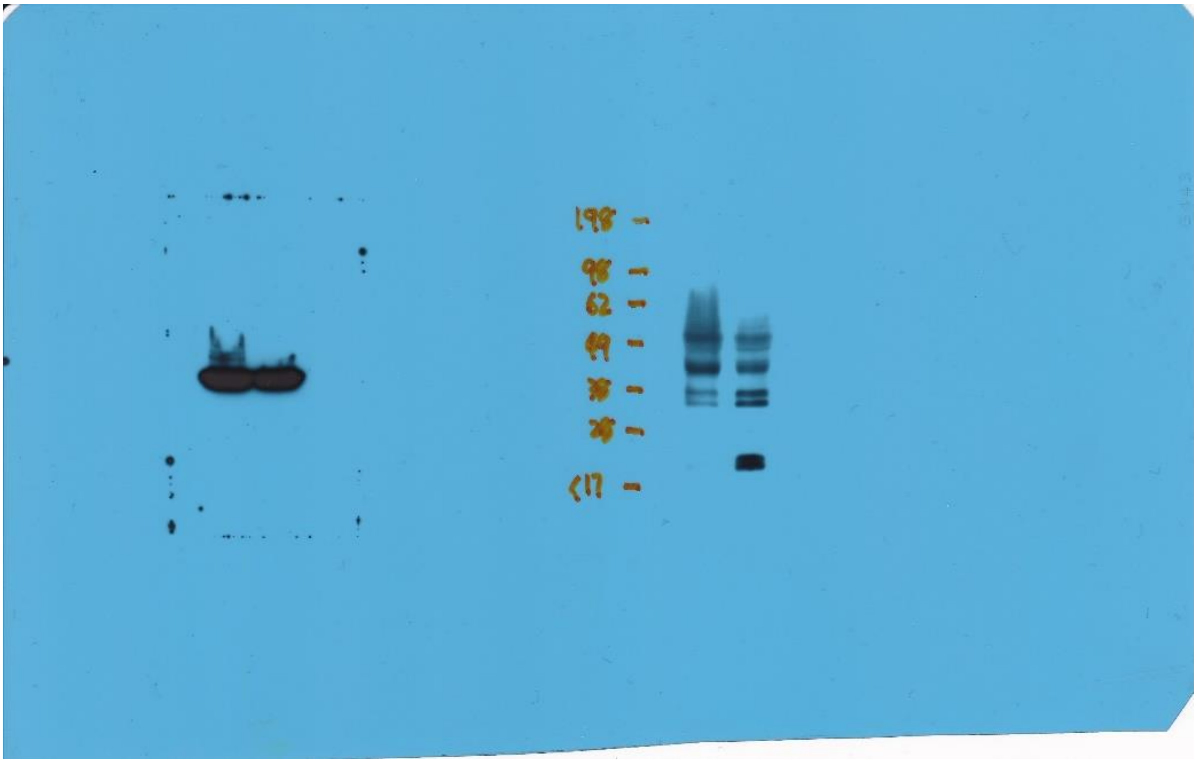

Full length uncropped original western blot of Figure 5e

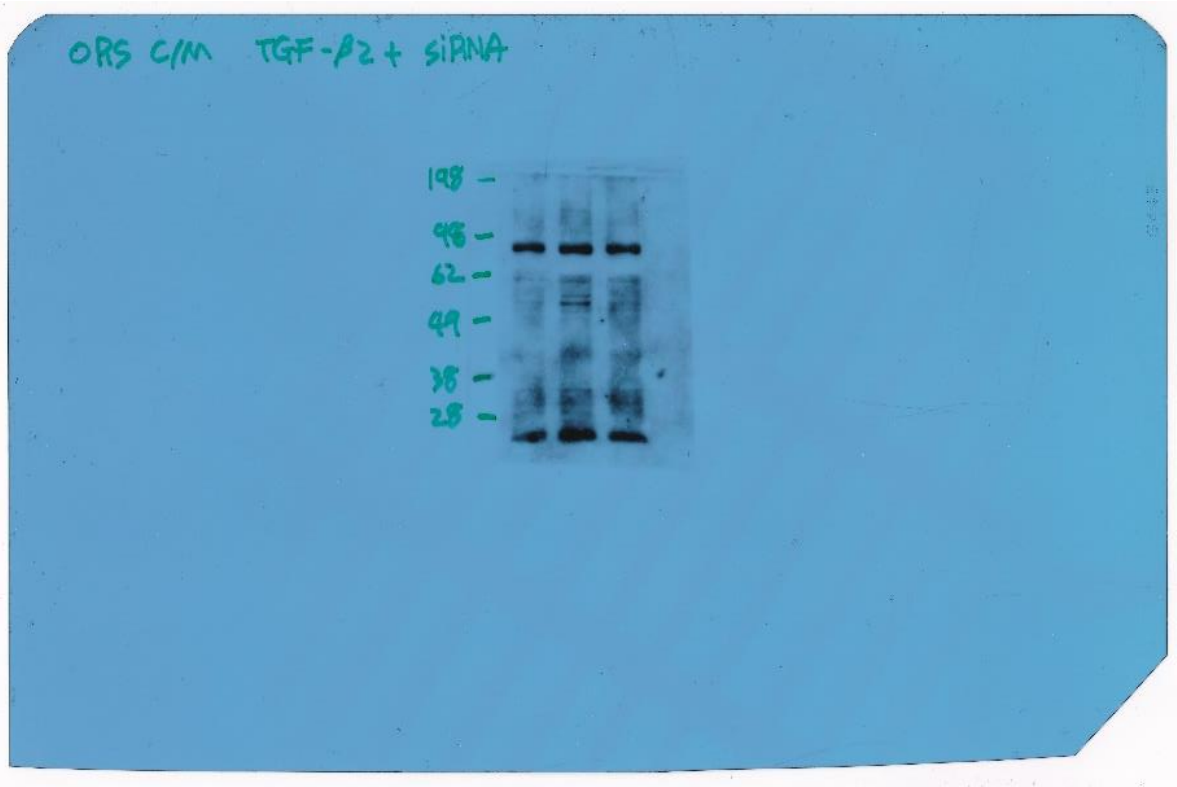

Full length uncropped original western blots of Figure 6a

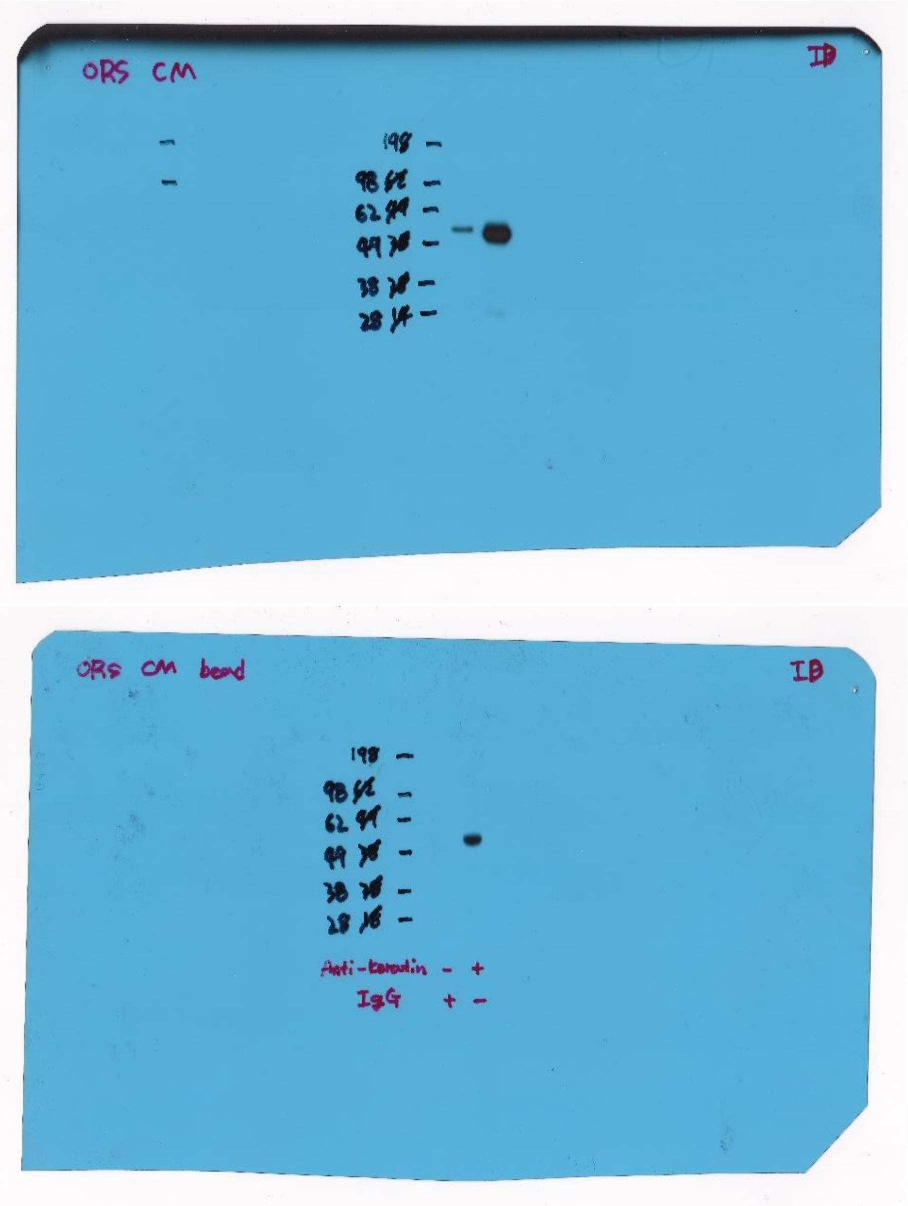

Full length uncropped original western blots of Figure 6d

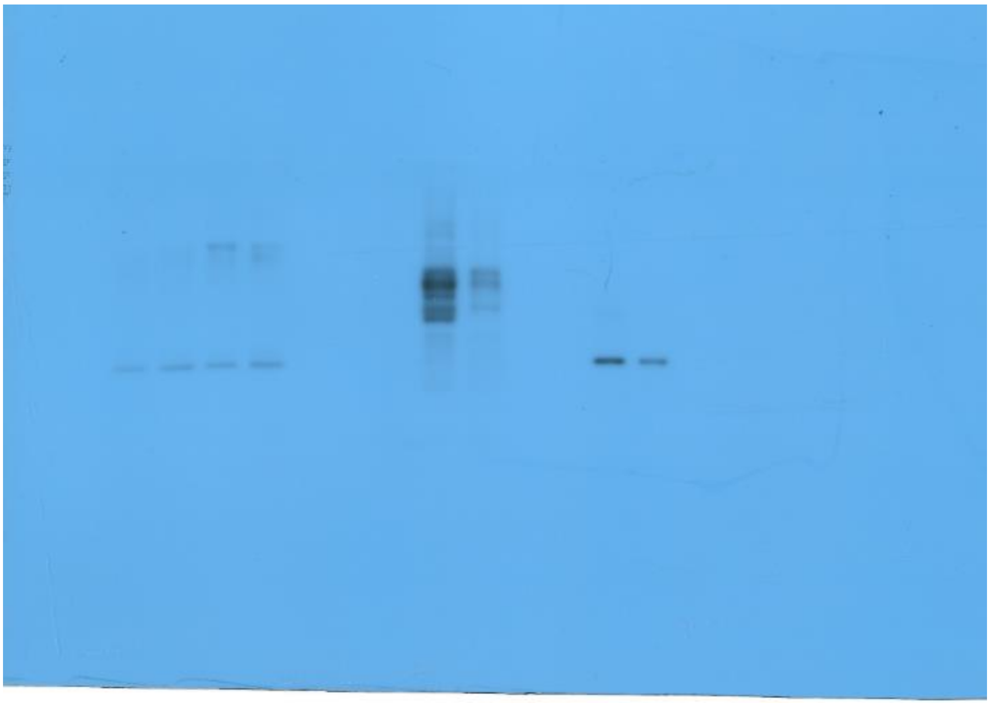

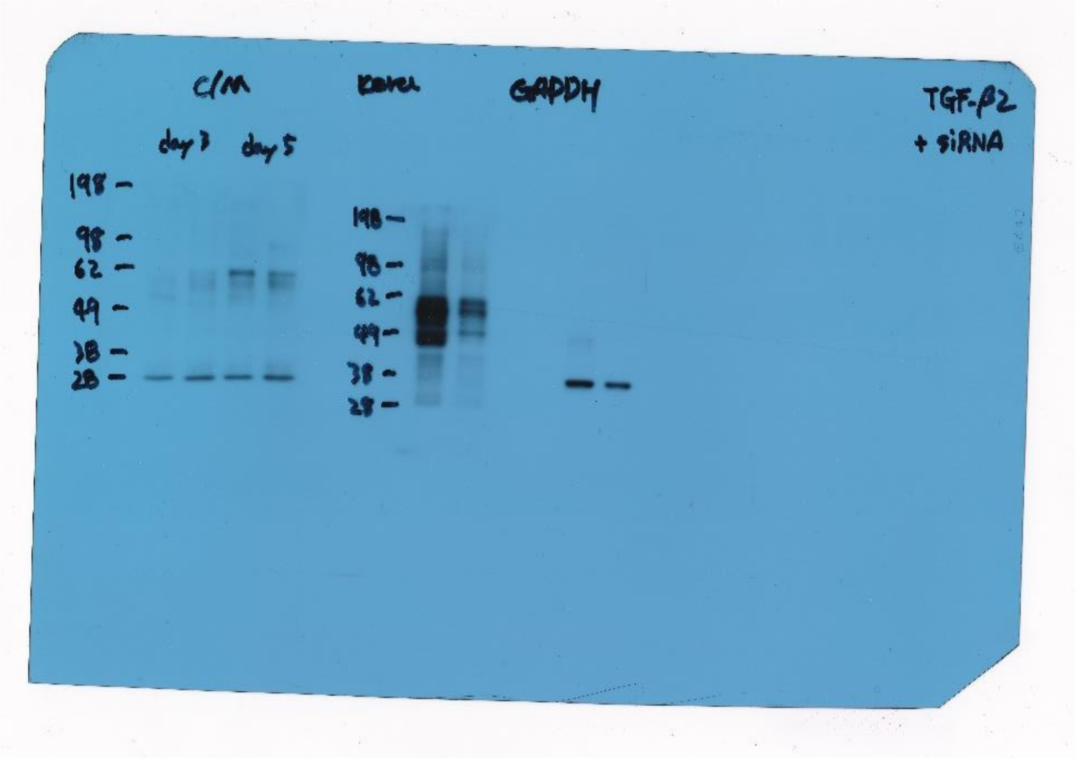

Full length uncropped original western blots of Supplementary Figure 18b

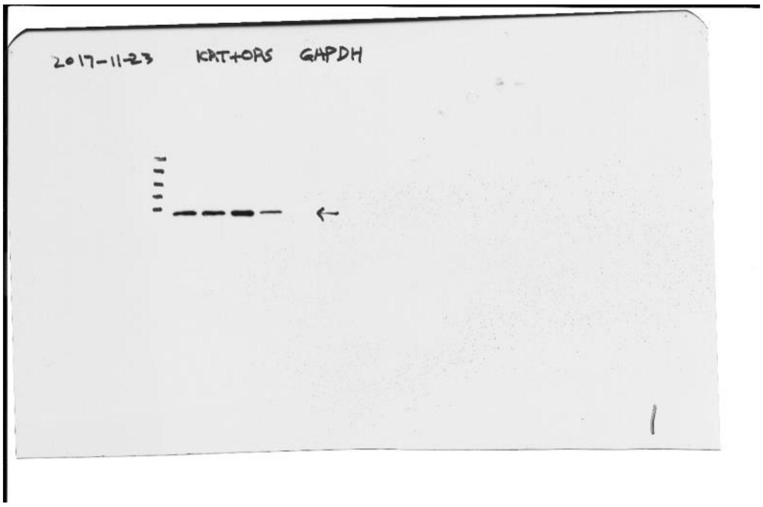

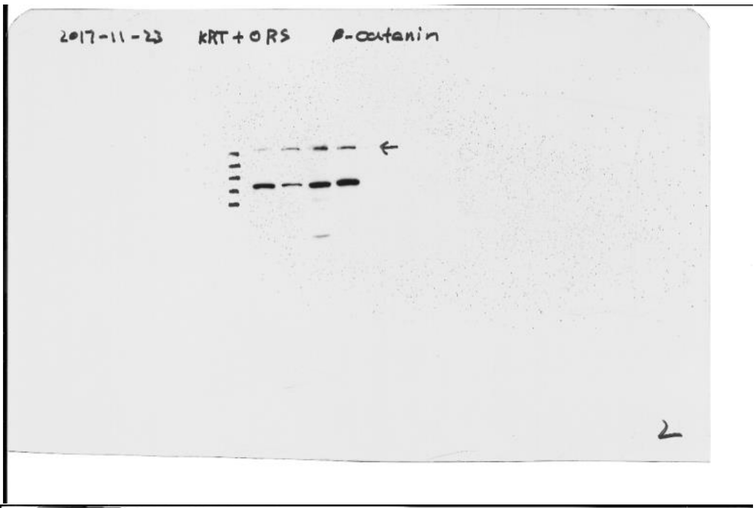

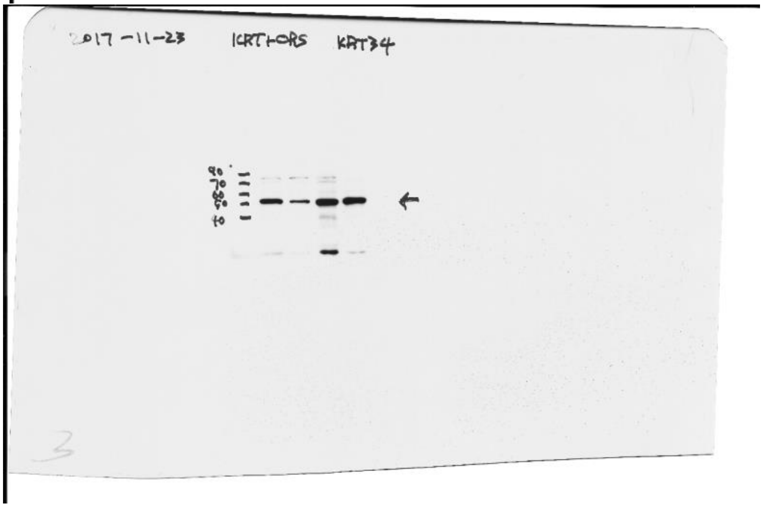

Full length uncropped original antibody array of Supplementary Figure 19

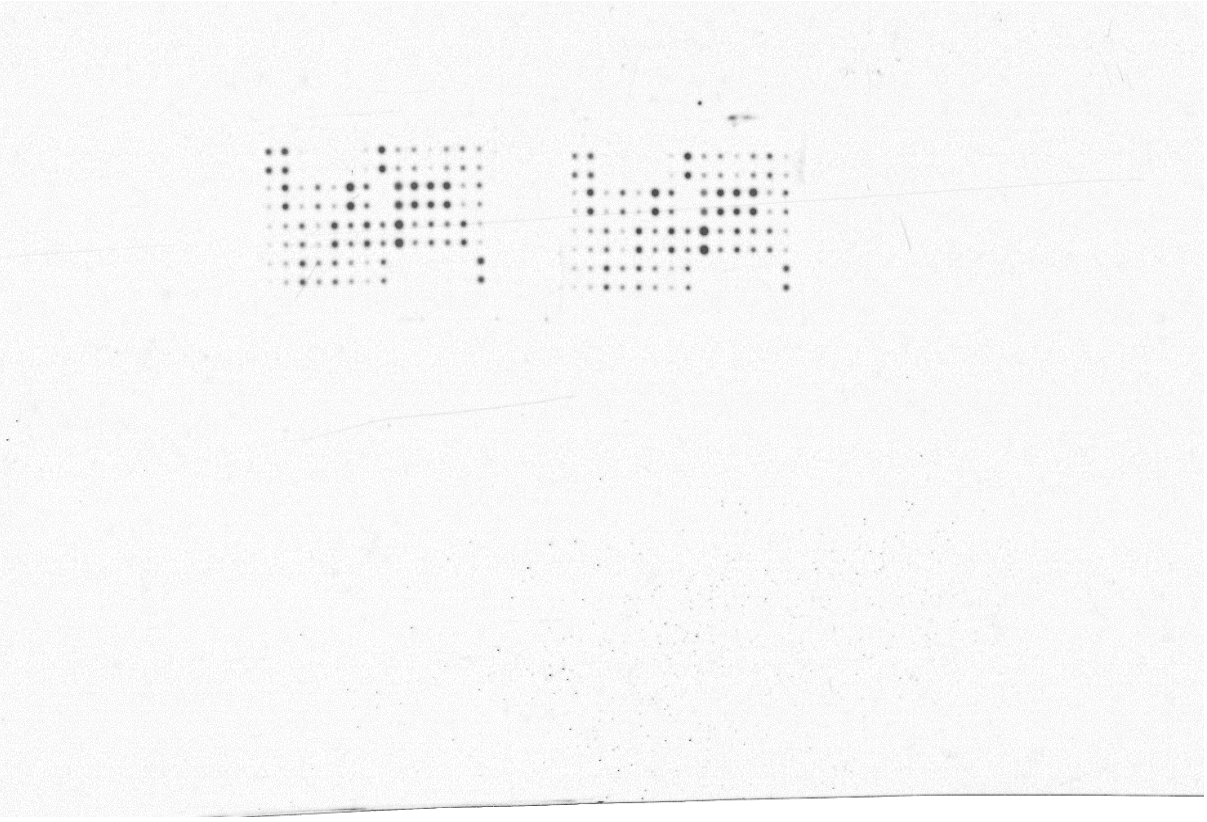

Full length uncropped original western blots of Supplementary Figure 20

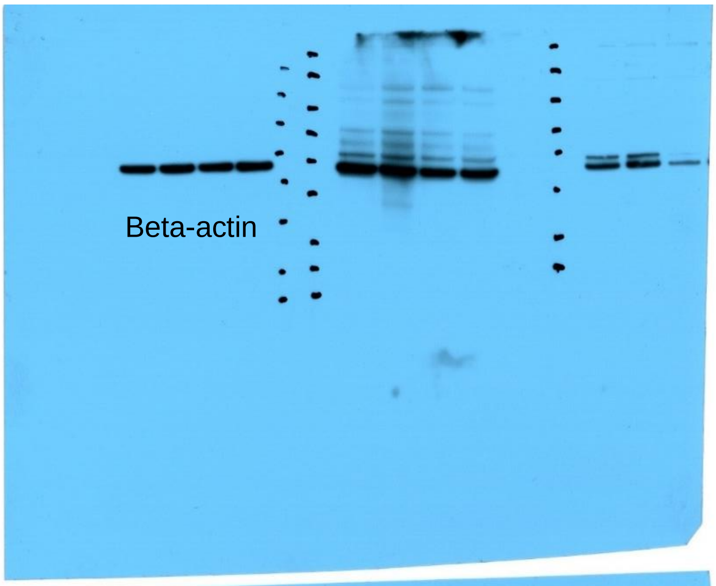

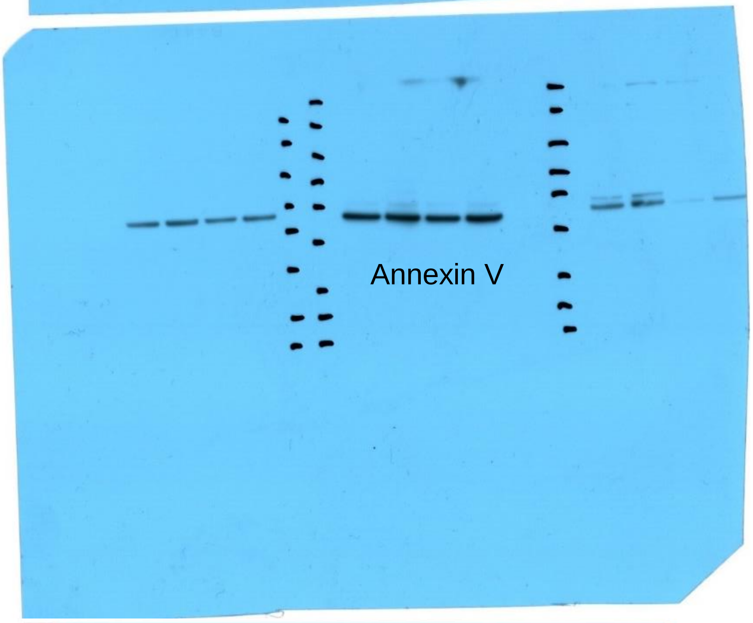

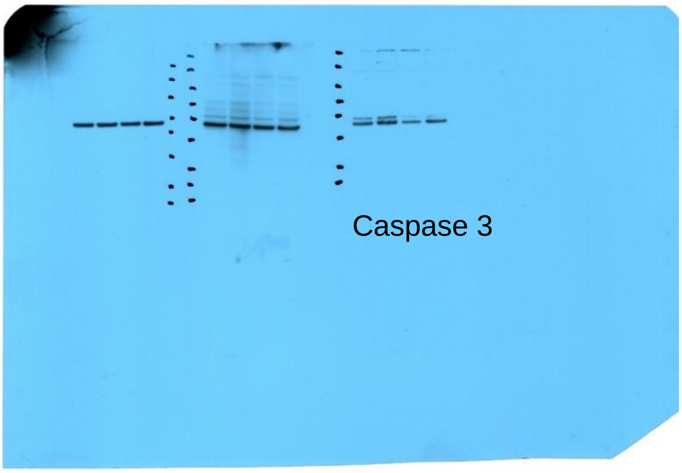

Full length uncropped original antibody array of Supplementary Figure 23

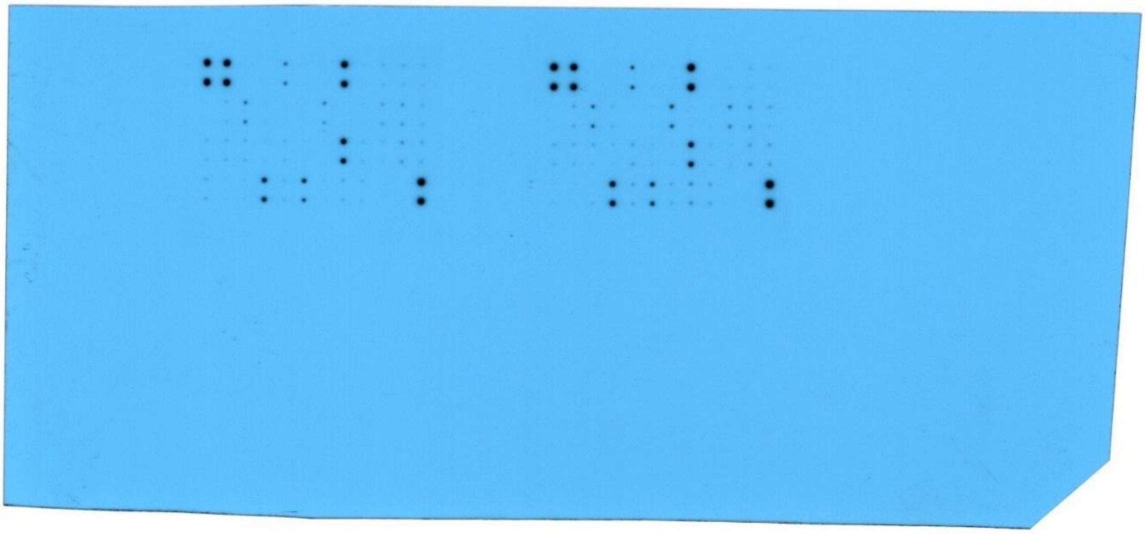

Full length uncropped original western blot and SDS-PAGE gel of Supplementary Figure 33

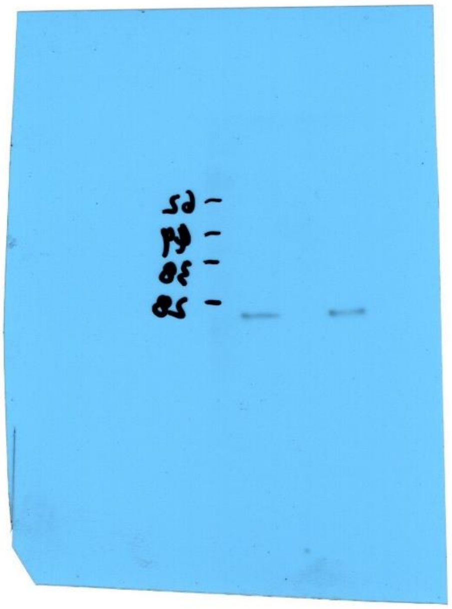

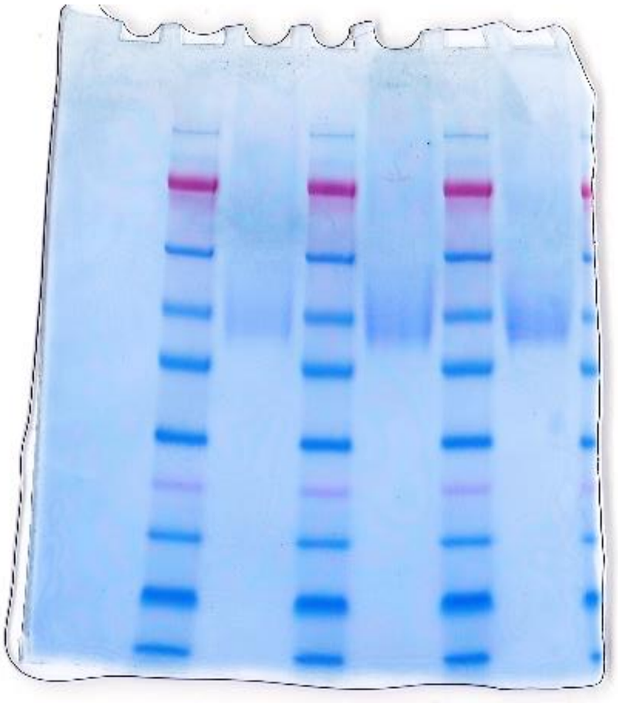

SUPPLEMENTARY TABLES

Table S1. Primers used in real time-qPCR

Forward *CD133* CAGAGTACAACGCCAAACCA	Reverse *CD133* AAATCACGATGAGGGTCAGC
Forward *Vesican* GGGATTGAAGACACACAAGACACG	Reverse *Versican* TGCTCTGGAGTTGCTATGACTGCC
Forward *Corin* GTACAGTGCGGTGTCCAACA	Reverse *Corin* ATCCTGTCAATCCTACCCCC
Forward *β-catenin* TGCAGTTCGCCTTCACTATG	Reverse *β-catenin* CTGCACAAACAATGGAATGG
Forward *SOX2* GCGGAGTGGAAACTTTTGTCC	Reverse *SOX2* CGGGAAGCGTGTACTTATCCTT
Forward *Shh* GATGTCTGCTGCTAGTCCTCG	Reverse *Shh* CACCTCTGAGTCATCAGCCTG
Forward Gapdh GGCTCTCCAGAACATCATCC	Reverse *Gapdh* TTTCTAGACGGCAGGTCAGG
Forward *TGFβ2* AAAGTGGACGTAGGCAGCAATTA	Reverse *TGFβ2* GACCAACCGGCGGAAGA
Forward *BMP6* CCTTCCCATCCTTTCTGCGA	Reverse *BMP6* CCGTCGCTCCCACTCAAAAT
Forward *FGF7* AGAAAGGCTCAAGTTGCACCA	Reverse *FGF7* GCTGTGACGCTGTTTGCTAT
Forward *FGF10* TTGTAGAAGTGGCTCGCAGG	Reverse *FGF10* ACAAAACCCAAGGGAGGTGG
Forward *hKRT3*1 TTCCTTCGTGCGCTAGAACC	Reverse *hKRT31* CAGGCCCCTTTTGTTGAACC
Forward *hKRT34* GAAGCATCTCAGGCTGGCATA	Reverse *hKRT34* GCAAGGGTGCTCAGAGGTATC
Forward *EDAR* CTCTGCCCCAGCCTGTTG	Reverse *EDAR* GCTTTGCTGGAGTTGCTGTC
Forward *FOXN1* AGTCCTGGGTTCAGAGGTCA	Reverse *FOXN1* CTGGCGAGTACTGGTGGAAG
Forward *EFNB1* TGCCTGTAGCACAGTTCTCG	Reverse *EFNB1* CTTGAACTCCAGGCCCATGT
Forward *MSX2* CCGCCAAGACATATGAGCCC	Reverse *MSX2* GGAGAGGTACTGTTTCTGACGG
Forward *ITGA6* GAAGGTGGCTGCGGTAGCA	Reverse *ITGA6* ATCACGTTGTCCTCCCGAGT
Forward *Sox9* GGACCACCCGGATTACAAGT	Reverse *Sox9* AAGATGGCGTTGGGGGAGAT

Table S2. Primers used in *in vivo* KRT31/KRT34 silencing study

Forward *mKrt31* CAAGCAATGCATGCGGCAA	Reverse *mKrt31* CGTACAAAGGAGTTGCATGGC
Forward *mKrt34* ATGTCCTGTGAGTCTTGCCTG	Reverse *mKrt34* GAACCAATTGCAGTTGCCCA
Forward *mGapdh* GGAGAGTGTTTCCTCGTCCC	Reverse *mGapdh* ATGAAGGGGTCGTTGATGGC
