# Supplementary material for: Keratin-mediated hair growth and its underlying biological mechanism
Source: Commun Biol. 2022 Nov 19;5:1270. doi: 10.1038/s42003-022-04232-9 (PMC9675858; doi:10.1038/s42003-022-04232-9)
Supplement: Supplementary file 3 — Description of Additional Supplementary Files [file 42003_2022_4232_MOESM3_ESM.pdf]

Description of Additional Supplementary Files

File name: Supplementary Data 1

Description: The source data behind the graphs (Figure 1c, 1d and1e) in the paper

File name: Supplementary Data 2

Description: The source data behind the graphs (Figure 2a and 2d) in the paper

File name: Supplementary Data 3

Description: The source data behind the graphs (Figure 4a) in the paper

File name: Supplementary Data 4

Description: The source data behind the graphs (Figure 5b, 5d and 5f) in the paper

File name: Supplementary Data 5

Description: The source data behind the graphs (Figure 6b and 6c) in the paper

File name: Supplementary Data 6

Description: The source data behind the graphs (Figure 7a, 7c and 7e) in the paper
