# Supplementary material for: Keratin-mediated hair growth and its underlying biological mechanism
Source: Commun Biol. 2022 Nov 19;5:1270. doi: 10.1038/s42003-022-04232-9 (PMC9675858; doi:10.1038/s42003-022-04232-9)
Supplement: Supplementary file 10 — Reporting Summary [file 42003_2022_4232_MOESM10_ESM.pdf]

nature portfolio

Corresponding author(s): COMMSBIO-20-3162C

Last updated by author(s): Oct 19, 2022

Reporting Summary

Nature Portfolio wishes to improve the reproducibility of the work that we publish. This form provides structure for consistency and transparency in reporting. For further information on Nature Portfolio policies, see our Editorial Policies and the Editorial Policy Checklist.

Statistics

For all statistical analyses, confirm that the following items are present in the figure legend, table legend, main text, or Methods section.

n/a	Confirmed	
☐	☒	The exact sample size (*n*) for each experimental group/condition, given as a discrete number and unit of measurement
☐	☒	A statement on whether measurements were taken from distinct samples or whether the same sample was measured repeatedly
☐	☒	The statistical test(s) used AND whether they are one- or two-sided<br>*Only common tests should be described solely by name; describe more complex techniques in the Methods section.*
☐	☒	A description of all covariates tested
☐	☒	A description of any assumptions or corrections, such as tests of normality and adjustment for multiple comparisons
☐	☒	A full description of the statistical parameters including central tendency (e.g. means) or other basic estimates (e.g. regression coefficient) AND variation (e.g. standard deviation) or associated estimates of uncertainty (e.g. confidence intervals)
☐	☒	For null hypothesis testing, the test statistic (e.g. *F*, *t*, *r*) with confidence intervals, effect sizes, degrees of freedom and *P* value noted<br>*Give P values as exact values whenever suitable.*
☒	☐	For Bayesian analysis, information on the choice of priors and Markov chain Monte Carlo settings
☒	☐	For hierarchical and complex designs, identification of the appropriate level for tests and full reporting of outcomes
☒	☐	Estimates of effect sizes (e.g. Cohen's *d*, Pearson's *r*), indicating how they were calculated

Our web collection on statistics for biologists contains articles on many of the points above.

Software and code

Policy information about availability of computer code

Data collection	*Provide a description of all commercial, open source and custom code used to collect the data in this study, specifying the version used OR state that no software was used.*
Data analysis	*Provide a description of all commercial, open source and custom code used to analyse the data in this study, specifying the version used OR state that no software was used.*

For manuscripts utilizing custom algorithms or software that are central to the research but not yet described in published literature, software must be made available to editors and reviewers. We strongly encourage code deposition in a community repository (e.g. GitHub). See the Nature Portfolio guidelines for submitting code & software for further information.

Data

Policy information about availability of data

All manuscripts must include a data availability statement. This statement should provide the following information, where applicable:

- Accession codes, unique identifiers, or web links for publicly available datasets
- A description of any restrictions on data availability
- For clinical datasets or third party data, please ensure that the statement adheres to our policy

All data that support the findings of this study are available from the corresponding author upon reasonable request. Full-length uncropped original western blots used in the manuscript are shown in Supplementary Figure 42. The numerical data that make up the all graphs in the paper are shown in Supplementary Data 1-6.

The transcriptome sequencing data (RNA-Seq) have been deposited at NCBI GenBank under BioProject ID PRJNA576064 (BioSample SAMN12924151 - SAMN12924158). The mass spectrometry proteomics data have been deposited to the ProteomeXchange Consortium via the PRIDE partner repository with the dataset identifier PXD-----.

Human research participants

Policy information about studies involving human research participants and Sex and Gender in Research.

Reporting on sex and gender	*Use the terms sex (biological attribute) and gender (shaped by social and cultural circumstances) carefully in order to avoid confusing both terms. Indicate if findings apply to only one sex or gender; describe whether sex and gender were considered in study design whether sex and/or gender was determined based on self-reporting or assigned and methods used. Provide in the source data disaggregated sex and gender data where this information has been collected, and consent has been obtained for sharing of individual-level data; provide overall numbers in this Reporting Summary. Please state if this information has not been collected. Report sex- and gender-based analyses where performed, justify reasons for lack of sex- and gender-based analysis.*
Population characteristics	*Describe the covariate-relevant population characteristics of the human research participants (e.g. age, genotypic information, past and current diagnosis and treatment categories). If you filled out the behavioural & social sciences study design questions and have nothing to add here, write "See above."*
Recruitment	*Describe how participants were recruited. Outline any potential self-selection bias or other biases that may be present and how these are likely to impact results.*
Ethics oversight	*Identify the organization(s) that approved the study protocol.*

Note that full information on the approval of the study protocol must also be provided in the manuscript.

Field-specific reporting

Please select the one below that is the best fit for your research. If you are not sure, read the appropriate sections before making your selection.

☒ Life sciences ☐ Behavioural & social sciences ☐ Ecological, evolutionary & environmental sciences

For a reference copy of the document with all sections, see nature.com/documents/nr-reporting-summary-flat.pdf

Life sciences study design

All studies must disclose on these points even when the disclosure is negative.

Sample size	The number of samples per independent experiment are described in the legends.
Data exclusions	*Describe any data exclusions. If no data were excluded from the analyses, state so OR if data were excluded, describe the exclusions and the rationale behind them, indicating whether exclusion criteria were pre-established.*
Replication	Most assays were repeated independently at least two times, and all values obtained from in vitro and in vivo analysis are presented as the mean ± standard deviation (SD). Statistically differences were identified by two-sided Student's t-test or one-way ANOVA parametric test. A P-value of less than 0.05 was considered significant.
Randomization	*Describe how samples/organisms/participants were allocated into experimental groups. If allocation was not random, describe how covariates were controlled OR if this is not relevant to your study, explain why.*
Blinding	*Describe whether the investigators were blinded to group allocation during data collection and/or analysis. If blinding was not possible, describe why OR explain why blinding was not relevant to your study.*

Reporting for specific materials, systems and methods

We require information from authors about some types of materials, experimental systems and methods used in many studies. Here, indicate whether each material, system or method listed is relevant to your study. If you are not sure if a list item applies to your research, read the appropriate section before selecting a response.

Materials & experimental systems

n/a	Involved in the study
☐	☒ Antibodies
☐	☒ Eukaryotic cell lines
☒	☐ Palaeontology and archaeology
☐	☒ Animals and other organisms
☒	☐ Clinical data
☒	☐ Dual use research of concern

Methods

n/a	Involved in the study
☒	☐ ChIP-seq
☐	☒ Flow cytometry
☒	☐ MRI-based neuroimaging

Antibodies

Antibodies used

rabbit anti-β-catenin (Abcam, ab16051, diluted 1:100)
rabbit anti-SOX2 (Cell Signaling Technology, 3579S, diluted 1:200)
rabbit anti-CD133 (Abcam, ab16518, diluted 1:50)
mouse anti-integrin β1 (Santa Cruz Biotechnology, sc-59829, diluted 1:50)
rabbit anti-P-cadherin (Cell Signaling Technology, 2189S, diluted 1:50)
mouse anti-E-cadherin (Abcam, ab1416, diluted 1:100)
mouse anti-alkaline phosphatase (Abcam, ab126820, diluted 1:100)
mouse anti-RUNX1 (Santa Cruz Biotechnology, sc-365644, diluted 1:50)
rabbit anti-KRT34 (LifeSpan BioSciences, LS-B15620, diluted 1:100)
rabbit anti-FGF7 (Santa Cruz Biotechnology, sc-7882, diluted 1:50)
goat anti-FGF10 (Santa Cruz Biotechnology, sc-7375, diluted 1:50)
goat anti-BMP6 (Santa Cruz Biotechnology, sc-7406, diluted 1:50)
rabbit anti-CD34 (Abcam, ab81289, diluted 1:100)
rabbit anti-SOX9 (Abcam, ab185966, diluted 1:100)
rabbit anti-Annexin V (Abcam, ab14196, diluted 1:100)
rabbit anti-caspase-3 (Abcam, ab13847, diluted 1:100)
rabbit anti-caspase-6 (Abcam, ab52951, diluted 1:100)
mouse anti-BrdU (Invitrogen, MA3-071, diluted 1:100)
rabbit anti-Ki67 (Cell Signaling Technology, 9027S, diluted 1:100)
rabbit anti-Lgr5 (Abcam, ab219107, diluted 1:100)
rabbit anti-Vinculin (Abcam, ab129002, diluted 1:100)
Alexa Fluor 488 conjugated goat anti-rabbit IgG (Invitrogen, A-11034, diluted 1:200)
Alexa Fluor 594 conjugated goat anti-rabbit IgG (Invitrogen, A-11012, diluted 1:200)
Alexa Fluor 594 conjugated goat anti-mouse IgG (Invitrogen, A-11032, diluted 1:200)
Alexa Fluor 488 conjugated goat anti-mouse IgG (Invitrogen, A-11001, diluted 1:200).
rhodamine phalloidin (Invitrogen, R415, diluted 1:400)
goat anti-P-cadherin (R&D Systems, AF761, diluted 1:50)
rabbit anti-β-catenin (Abcam, ab16051, diluted 1:50)
rabbit anti-KRT34 (Biorbyt, orb628339, diluted 1:100)
guinea pig anti-type I+II hair keratins (PROGEN, GP-panHK, diluted 1:50)
mouse anti-Ki67 (Abcam, ab279653, diluted 1:50)
rabbit anti-Annexin V (Abcam, ab14196, diluted 1:100)
rabbit anti-Caspase-3 (Abcam, ab13847, diluted 1:50),
rabbit anti-active Caspase-3 (Abcam, ab32042, diluted 1:50).
donkey Alexa Fluor 488-conjugated anti-goat IgG (Invitrogen, A-11055, diluted 1:200)
goat Alexa Fluor 594-conjugated anti-rabbit IgG (Invitrogen, A-11012, diluted 1:200)
goat Alexa Fluor 488-conjugated anti-rabbit IgG (Invitrogen, A-11034, diluted 1:200)
goat Alexa Fluor 647-conjugated anti-guinea pig IgG (Abcam, ab150187, diluted 1:200)

Validation

All antibodies used in this study were purchased, and the companies were as followes; Ab Cam, Santa Cruz Biotechnology, Cell Signaling Technology, Invitrogen, R&D systems, Biorbyt, Progen.

Eukaryotic cell lines

Policy information about cell lines and Sex and Gender in Research

Cell line source(s)

Human outer root sheath cells (ORS; CEFO, CB-ORS-001: 36 years old female) and human dermal papilla cells (DP; CEFO, CB-HDP-001: 54 years old male)

Authentication

All cells used in this study were commercially available and purchased from Cell Bio company (https://cefobio.com/).

Mycoplasma contamination

The Cel-bio company tested virus and mycoplasma contaminations prior to selling

Commonly misidentified lines
(See ICLAC register)

Name any commonly misidentified cell lines used in the study and provide a rationale for their use.

Animals and other research organisms

Policy information about studies involving animals; ARRIVE guidelines recommended for reporting animal research, and Sex and Gender in Research

Laboratory animals	six-week-old C57BL/6 male mice
Wild animals	*Provide details on animals observed in or captured in the field; report species and age where possible. Describe how animals were caught and transported and what happened to captive animals after the study (if killed, explain why and describe method; if released, say where and when) OR state that the study did not involve wild animals.*
Reporting on sex	*Indicate if findings apply to only one sex; describe whether sex was considered in study design, methods used for assigning sex. Provide data disaggregated for sex where this information has been collected in the source data as appropriate; provide overall numbers in this Reporting Summary. Please state if this information has not been collected. Report sex-based analyses where performed, justify reasons for lack of sex-based analysis.*
Field-collected samples	The animals were housed in a room that was maintained at a temperature of 23 ± 3 ℃ and a relative humidity of 55 ± 15 %, with artificial lighting from 08:00 to 20:00, 150-300 Lux of luminous intensity. Throughout the experimental period, the temperature and humidity of animal room were measured every hour with a computer-based automatic sensor, and as a result of measurements, there were no deviations to have adverse effect to the result of study.
Ethics oversight	Animal experiments were approved by the Institutional Animal Care and Use Committee of Konkuk University (KU18159, KU19066), and procedures on animals were performed in accordance with the relevant guidelines and regulations.<br>Animals experiment were performed based on standard operating procedure of Chemon.Inc and the Animal experimentation policy of Gyeonggi bio center (experimentation number: 2018-08-011).

Note that full information on the approval of the study protocol must also be provided in the manuscript.

Flow Cytometry

Plots

Confirm that:

☒ The axis labels state the marker and fluorochrome used (e.g. CD4-FITC).

☒ The axis scales are clearly visible. Include numbers along axes only for bottom left plot of group (a 'group' is an analysis of identical markers).

☒ All plots are contour plots with outliers or pseudocolor plots.

☐ A numerical value for number of cells or percentage (with statistics) is provided.

Methodology

Sample preparation	ORS cells were cultured in the presence of TGF[illegible]2 and harvested by incubating with 0.25% Trypsin/EDTA for 10 min at 37 ℃. Cells were recovered by centrifugation and fixed with 0.01% formaldehyde in DPBS for 15 min at room temperature.
Instrument	FACSCanto (BD Biosciences, USA)
Software	FlowJo V10 program
Cell population abundance	*Describe the abundance of the relevant cell populations within post-sort fractions, providing details on the purity of the samples and how it was determined.*
Gating strategy	*Describe the gating strategy used for all relevant experiments, specifying the preliminary FSC/SSC gates of the starting cell population, indicating where boundaries between "positive" and "negative" staining cell populations are defined.*

☐ Tick this box to confirm that a figure exemplifying the gating strategy is provided in the Supplementary Information.
